# Sexually Dimorphic Response to Hepatic Injury in Newborn Suffering from Intrauterine Growth Restriction

**DOI:** 10.1002/advs.202403095

**Published:** 2024-06-13

**Authors:** Yu‐Sen Wei, Wen‐Jie Tang, Pei‐Yu Mao, Jiang‐Di Mao, Zhi‐Xiang Ni, Kang‐Wei Hou, Teresa G. Valencak, Da‐Ren Liu, Jun‐Fang Ji, Hai‐Feng Wang

**Affiliations:** ^1^ College of Animal Science Zhejiang University The Key Laboratory of Molecular Animal Nutrition Ministry of Education Hangzhou 310000 China; ^2^ Department of Gynecology and Obstetrics The First Affiliated Hospital of Zhejiang Chinese Medical University (Zhejiang Provincial Hospital of Chinese Medicine) Hangzhou 310006 China; ^3^ The Second Affiliated Hospital of Zhejiang University Hangzhou 310009 China; ^4^ The MOE Key Laboratory of Biosystems Homeostasis & Protection Life Sciences Institute Zhejiang University Hangzhou 310058 China

**Keywords:** hepatic injury, intrauterine growth restriction, piglet, sexual dimorphism, single‐cell RNA sequencing

## Abstract

Intrauterine growth restriction (IUGR), when a fetus does not grow as expected, is associated with a reduction in hepatic functionality and a higher risk for chronic liver disease in adulthood. Utilizing early developmental plasticity to reverse the outcome of poor fetal programming remains an unexplored area. Focusing on the biochemical profiles of neonates and previous transcriptome findings, piglets from the same fetus are selected as models for studying IUGR. The cellular landscape of the liver is created by scRNA‐seq to reveal sex‐dependent patterns in IUGR‐induced hepatic injury. One week after birth, IUGR piglets experience hypoxic stress. IUGR females exhibit fibroblast‐driven T cell conversion into an immune‐adapted phenotype, which effectively alleviates inflammation and fosters hepatic regeneration. In contrast, males experience even more severe hepatic injury. Prolonged inflammation due to disrupted lipid metabolism hinders intercellular communication among non‐immune cells, which ultimately impairs liver regeneration even into adulthood. Additionally, Apolipoprotein A4 (APOA4) is explored as a novel biomarker by reducing hepatic triglyceride deposition as a protective response against hypoxia in IUGR males. PPARα activation can mitigate hepatic damage and meanwhile restore over‐expressed APOA4 to normal in IUGR males. The pioneering study offers valuable insights into the sexually dimorphic responses to hepatic injury during IUGR.

## Graphical abstract

Intrauterine growth restriction (IUGR) induced by hypoxia leads to sex‐specific liver injury. In IUGR females, a slight inflammatory response occurs due to fibroblast‐driven T cells converting to an immune‐adapted phenotype. In IUGR males, excessive inflammation driven by macrophages is caused by triglyceride deposition. The protective upregulation of apolipoprotein apolipoprotein A4 (APOA4) in hepatocytes helps IUGR males survive.

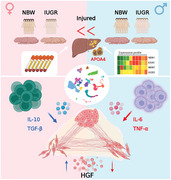

## 1 Introduction

The hypothesis of “Fetal Origin of Adult Disease” has developed into the theory of “Developmental origin of health and disease”. This concept consistently suggests that adverse events during pregnancy resulting in low birth weight (LBW), as well as early environmental factors in the postnatal stage, are linked to a higher risk of chronic diseases during adulthood. To date, this paradigm has been extended to several metabolic diseases.^[^ ^1^ ^]^ The liver is highlighted as one of the target organs and undergoes structural and functional changes in response to adverse intrauterine conditions.^[^ ^2^ ^]^

In 1961, the WHO established the criterion for human LBW as a birth weight below 2500 g, regardless of gestational age or maturity. Intrauterine growth restriction (IUGR) refers to a condition in which the fetus fails to reach its intended growth potential and is often characterized by a birth weight two standard deviations below the mean or below the 10th percentile.^[^ ^3^ ^]^ IUGR has been associated with various pregnancy disorders, most notably intrauterine hypoxia.^[^ ^4^ ^]^ The incidence of IUGR varies from 4–8% in industrialized countries to 30% in less developed countries.^[^ ^3^ ^]^ Given that perinatal and early postnatal environments open specific windows for fatal reprogramming,^[^ ^5^ ^]^ there is growing evidence that intrauterine hypoxia, resulting from multiple factors, could lead to long‐term negative alterations in offspring.^[^ ^4^ ^]^ The liver is a potential organ for programming and acts as a crucial site for insulin‐like growth factor I (IGF‐1) synthesis, stimulating systemic body growth.^[^ ^6^ ^]^ Hence, it is necessary to study and understand the reprogramming of the liver in response to IUGR. Additionally, there is a clear sex difference in hepatic metabolism from birth,^[^ ^7^ ^]^ with males being more prone to hepatic problems during adulthood.^[^ ^8^ ^]^ Yet, there is limited knowledge on sex‐specific interventions for IUGR.

In our study, based on previous transcriptome investigations,^[^ ^9^ ^]^ we conducted single‐cell RNA sequencing (scRNA‐seq) on hepatic samples obtained from normal birth weight (NBW) and IUGR piglets one week after birth. Pigs serve as excellent models for translational medicine studies, and IUGR in pigs closely mimics the human situation when no medication is used but nutritional deficit intervention is used.^[^ ^10^ ^]^ Interestingly, by integrating the biochemical profiles of neonatal human infants and piglets, we observed a very similar alteration pattern in the indices of hepatic function caused by LBW in both males and females. With respect to the specific time points of organism plasticity, the pig model effectively compensates for the lack of human early clinical liver samples related to IUGR. Also, the high genetic and environmental resemblance among pig siblings allowed us to disentangle several effects during IUGR in the liver. Previous research has mostly focused on tissue‐level transcriptomics and proteomics, and numerous hypotheses have been proposed.^[^ ^11^ ^]^ Precise mechanisms by which IUGR‐induced hepatic injury leads to chronic liver disease remain to be elucidated. scRNA‐seq, which enables unbiased identification of cell types in complex tissues, has revolutionized our understanding of cellular diversity and function during health and disease, and has been applied to various research areas in hepatology,^[^ ^12^ ^]^ including non‐alcoholic fatty liver disease and fibrosis.

Here, NBW and IUGR piglets were selected from pairs at the same sex from the same fetus. We present data on transcriptional profiles of 41,969 high‐quality cells obtained from hepatic samples of NBW and IUGR individuals. These profiles exhibited strong homology with human‐derived liver single‐cell datasets. Notably, male livers experienced more severe injury and inflammation in response to IUGR compared with female counterparts one week postnatally. This apparent sex difference prompted us to investigate the factors underlying the enhanced capacity for hepatic regeneration observed in female IUGR individuals, as well as to explore the multi‐dimensional hepatic injury observed in male IUGR individuals. In females with IUGR, an anti‐inflammatory environment created by fibroblast‐driven T cell conversion led to an immune‐adapted phenotype that enhanced hepatic regeneration, potentially due to the up‐regulation of estradiol (E2). In contrast, in males with IUGR, increased macrophage recruitment and higher inflammation were triggered by hypoxia‐induced hepatocellular damage accompanied by triglyceride (TG) accumulation. The communication network of hepatic non‐immune cells was disrupted, which eventually impaired hepatic regeneration signals, persisting into adulthood. Moreover, through the construction of the apolipopretein A4 (APOA4)‐KO HepG2 cell line, we confirmed that the up‐regulation of APOA4 might serve as a protective mechanism against hypoxic stress in males suffering from IUGR. PPARα activation with an agonist alleviated hepatic damage and at the same time, restored the overexpression of APOA4 to normal levels in males suffering from IUGR. Therefore, APOA4 could potentially serve as a promising biomarker for detecting IUGR‐induced hepatic damage. Our study supports Barker's hypothesis and emphasizes a possible link between hepatic injury caused by IUGR and the increasing incidence of metabolic disorders in men. These findings may provide a new theoretical framework and strategic approach for screening IUGR‐induced hepatic injury and developing gender‐specific effective therapies.

## 2 Results

### 2.1 Biochemical Profile and Re‐Analysis of Pertinent Liver RNA‐seq Data Confirm IUGR‐Related Hepatic Damage in Males

**Figure 1:**
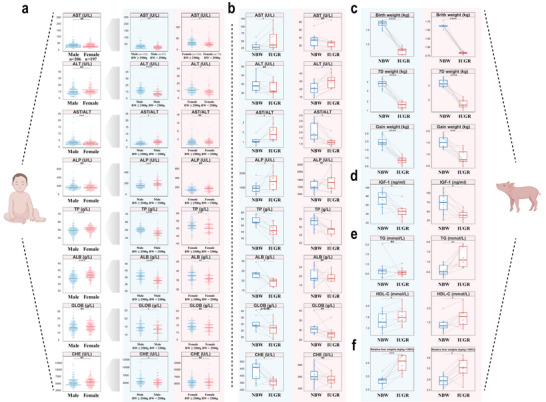
Biochemical parameters of infants and piglets showing a sex‐specific difference in liver function experiencing IUGR. a) Liver function indices (n = 73–206) (AST, ALT, ALP, TP, ALB, GLOB, CHE) in neonatal infants categorized by both sexes (male or female) and birth weight (low or normal). b–f) Liver function serum indices (AST, ALT, ALP, TP, ALB, GLOB, CHE) (b), growth performance (c), the concentration of IGF‐1 (d), TG and HDL‐C (e), and relative liver weights (f) in NBW and IUGR piglets of both sexes (n = 8). Unpaired Wilcoxon rank‐sum test (a) and two‐sided paired Student's t‐test (b–f) (****P < 0.0001; ***P < 0.001; **P < 0.01; *P < 0.05; ns *P* > 0.05). ALB, albumin; ALP, alkaline phosphatase; ALT, alanine aminotransferase; AST, aspartate transaminase; TP, total protein; CHE, cholinesterase; GLOB, globulin; HDL‐C, high‐density lipoprotein cholesterol. Male: blue background; Female: red background.

Gender‐specific differences were observed in the biochemical profiles of newborn infants (**Figure** **1**a; Table S1, Supporting Information). Compared with males, females exhibited significantly higher levels of total protein (TP) and albumin (ALB) but a lower aspartate aminotransferase/alanine aminotransferase (AST/ALT) ratio. Further classifications based on birth weights (<2500 g and ≥2500 g) led to new interesting findings for both genders. Both TP and ALB levels were lower in the LBW group than in the normal group in both sexes. However, in males, alkaline phosphatase (ALP) and AST/ALT increased, whereas globulin (GLOB) and cholinesterase (CHE) decreased in the LBW group compared with those in the normal group. These observations were not confirmed in females.

Serum parameters of the NBW and IUGR piglets, which were paired within the same fetus and separated by sex, showed similar trends observed in human LBW and normal infants by paired analysis (Figure 1b). IUGR piglets had considerably reduced growth capacity, characterized by lower birthweights, weights after one week, and overall weight gain in comparison to NBW piglets (Figure 1c). Reduced IGF‐1 levels were evident in all IUGR piglets (Figure 1d). High‐density lipoprotein cholesterol was notably upregulated in IUGR individuals, while changes in TG levels was differed in male and female IUGR individuals (Figure 1e). We did not observe any significant changes in low‐density lipoprotein cholesterol, total cholesterol, total biliary acid, total bilirubin, glucose or lactic dehydrogenase (Figure S1a–c, Supporting Information). With respect to organs, hepatic indices showed altered degrees of elevation in male and female individuals with IUGR compared with the corresponding NBW piglets (Figure 1f). Similarly, renal and pulmonary indices were significantly different in IUGR males (Figure S1d, Supporting Information).

A re‐analysis of the RNA‐seq data from PRJNA597972^[^ ^9a^ ^]^ aligned with the findings reported by the authors (Table S2, Supporting Information). One week after birth, males had greater transcriptional disorders than females suffering from IUGR. IUGR males had a total of 1074 differentially expressed genes (DEGs), as opposed to just 192 DEGs observed in IUGR females (Figure S2a, Supporting Information). There were intersections of 13 upregulated genes and 5 downregulated genes. Quantitative PCR with reverse transcription (RT‐qPCR) corroborated the expression of EPO and GAPDH was increased in IUGR individuals one week after birth (Figure S2b, Supporting Information). Functional enrichment analysis of the up‐ and down‐regulated genes using the Kyoto Encyclopedia of Genes and Genomes (KEGG) database revealed significant upregulation of the HIF‐1 signaling pathway in both sexes, while the TNF signaling pathway displayed the opposite trend (Figure S2c,d, Supporting Information). The presence of sex‐specific differences in IUGR‐related hepatic injury and inflammation, emphasized intrauterine hypoxia as the primary etiological factor for IUGR and points out distinct functional mechanisms in males and females. To further explore the impact of impaired transcription in IUGR males during adulthood, we reanalyzed the data from GSE106512^[^ ^9b^ ^]^ and focused on the difference between NBW and IUGR pigs at the age of 150 days. The DEGs from the early and adult stages were plotted onto chromosomal regions, and their intersections were counted (Figure S2e, Supporting Information). Persistent, aberrant metabolism, particularly about lipid metabolism, was found in the livers of IUGR males. Certain signal transduction processes, including proliferation, differentiation and immunological modulation, were perturbed during infancy, while disruptions in the cell cycle and Hippo signaling pathway became apparent in adulthood (Figure S2d, Supporting Information).

An analysis of pasture litter data suggested a marginally higher incidence of IUGR in males (Figure S2f, Supporting Information). Based on that, we conducted scRNA‐seq and lipidomics to understand the complex molecular mechanisms underlying sexual dimorphism during hepatic injury from IUGR (Figure S3, Supporting Information).

### 2.2 High Homology of Liver Comprehensive Single‐Cell Transcriptional Profile between Piglets and Humans

**Figure 2:**
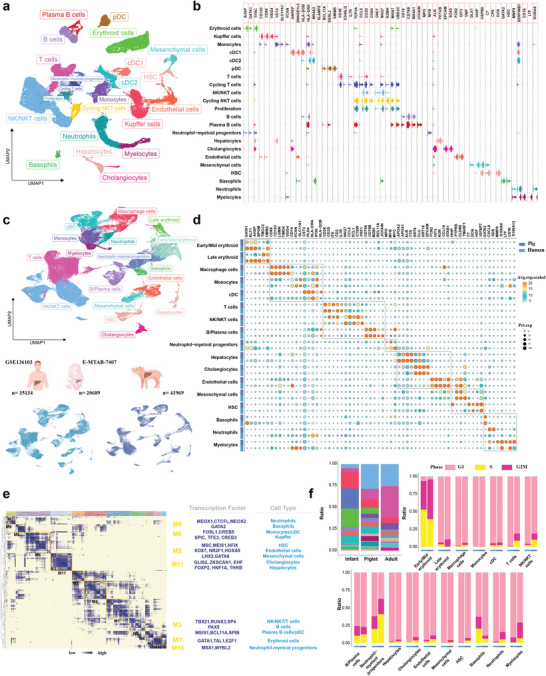
Overview of the hepatic cellular landscape of piglets and its homology to humans. a) UMAP plots illustrating cells from eight samples profiled in this study, colored by cluster assignment. b) Stacked violin plots presenting expression phenotypes of markers in these clusters. c–f) Integrated scRNA‐seq analysis, showcasing overlapping hepatic cell populations in UMAP plots (c), representative conserved signature genes in each cell type (d), similar transcriptional regulatory programs (e), and the status of cell cycle in each cell type (f) between pigs and humans.

A total of eight liver tissue samples obtained from normal and IUGR piglets, from males (n = 3) and females (n = 1) from each group, were collected for scRNA‐seq. We applied enzymatic dissociation to generate single‐cell suspensions and used the 10x Genomics platform for scRNA‐seq. In total, we sequenced 46,944 cells, achieving an average sequencing depth of 62,523 reads per cell, a sequencing saturation rate of 81%, and we detected an average of 968 genes per cell (Table S3, Supporting Information). After removing ambient mRNAs and applying stringent quality filtering based on the number of detected genes and mitochondrial read counts, a high‐quality dataset of 41,969 cells was retained for visualization of the correlation between QC‐related parameters (Figure S4a,b, Supporting Information). Graph‐based clustering was performed to group cells according to their gene‐expression profiles, and uniform manifold approximation and projection (UMAP) plots were used for visualization. Louvain clustering of these cells identified 22 major clusters representing diverse cell populations, including lymphoid, myeloid, epithelial, endothelial, fibroblast, erythroid populations and granulocytes (**Figure** **2**a). Cluster‐specific (Table S3, Supporting Information) and classic markers were used for accurate cluster annotation and cell type validation (Figure 2b), including T cells (CD3E; IL7R; CD40LG), NK/NKT cells (GNLY; NKG7; CD3D), B cells (CD79B; MS4A1; CD19), monocytes (CD14), Kupffer cells (CD68; CD163; VSIG4), endothelial cells (FCN2; OIT3; VWF), hepatocytes (ALB), HSC (DCN), mesenchymal cells (HHIP; DLK1), and neutrophils (MMP9). Scoring and displaying gene sets on UMAP plots were used to ensure the accuracy of cell identification (Figure S4c, Supporting Information).

To ascertain the degree of conservation or divergence in cellular architecture and molecular characteristics between pigs and humans, we integrated single‐cell data from healthy adult and fetal livers from GSE136103^[^ ^13^ ^]^ and E‐MTAB‐7407,^[^ ^14^ ^]^ respectively. By using homologous gene‐expression profiles for Louvain clustering and UMAP plots for visualization, we found that there was substantial overlap between human and porcine cell subpopulations (Figure 2c). The expression patterns of classic markers used to group cells into seven primary lineage compartments were strikingly similar in both species, spanning both immune and non‐immune cells (Figure 2d). The high homology of macrophages confirmed the advantage of porcine‐derived macrophage lines over murine‐derived ones for translational studies in human pathophysiology.^[^ ^15^ ^]^ Moreover, through modular clustering using the porcine cellular transcriptome, we found transcription factors regulating cellular differentiation in porcine liver were consistent with those in humans (Figure 2e; Table S4, Supporting Information). For instance, HNF1A and THRB promote the production of more mature hepatocytes;^[^ ^16^ ^]^ PAX5 controls B cells;^[^ ^17^ ^]^ GATA1 and TAL1 regulate the maturation of erythroid cells;^[^ ^18^ ^]^ SOX7 is closely linked to endothelial transformation;^[^ ^19^ ^]^ RUNX3 and TBX21 play pivotal roles in the growth and polarization of T cells.^[^ ^20^ ^]^ Despite different proportions of major cell types between human liver data from different time points, the distribution of cell cycle phases in each cell type was very similar between the two species (Figure 2f). These findings collectively indicate that pig livers act as meaningful models for exploring human hepatic disorders.

### 2.3 IUGR Males and Females Exhibit Distinct Levels of Cellular Transcription

To comprehensively examine the impact of IUGR on different cell types in both female and male subjects, UMAP plots were created to visualize cellular composition (Figure S5a,b, Supporting Information). In terms of the cell ratio, both IUGR females and males showed a significant increase in erythroid cells. This finding suggests that IUGR leads to a considerable developmental delay, as the liver is the site of hematopoiesis during the early stages of development.^[^ ^21^ ^]^ In females, the ratios of NK/NKT cells, T cells, monocytes and Kupffer cells were altered to some extent (Figure S5c, Supporting Information). In males, the number of Kupffer cells and NK/NKT cells was increased in IUGR piglets, as shown by the volcano plots, regardless of the inclusion of erythroid cells (Figure S5d, Supporting Information). However, sample size, the number of captured cells, and other factors may cause preferential changes in the ratio of a particular cell type.^[^ ^22^ ^]^ Since it may be misleading to draw conclusions based on cell proportions only, we examined and visualized DEGs in major cell types and compared enriched functions using Gene Ontology (GO) analysis. In IUGR females, there were more DEGs related to NK/NKT cells, monocytes, and T cells than to other cell types (Figure S6a, Supporting Information). These DEGs were associated with functional alterations related to cytokine stimulus responses, cell activation, the regulation of cellular defense, stress, cell adhesion, and inflammatory response (Figure S6b; Table S5, Supporting Information). Non‐immune cells in IUGR females suggested minimal changes, which might indicate an adaptation of the immune system to the effects of IUGR at a specific time point. In contrast, in IUGR males, there were more DEGs related to Kupffer cells, endothelial cells and hepatocytes than to other cell types (Figure S6c, Supporting Information). The DEGs in Kupffer cells were enriched in macrophage activation pathways, which are known to induce chronic inflammation,^[^ ^23^ ^]^ and are involved in cell death, apoptosis and reactive oxygen species (Figure S6d; Table S6, Supporting Information). Functional changes in cellular responses of lipids suggested a potential relationship between lipids and the activation of macrophages. Additionally, IUGR males exhibited impaired vascular development and angiogenesis in endothelial cells, as well as altered ketone metabolism and decreased detoxification in hepatocytes. Overall, lymphoid cells were most prominently modified by IUGR in females, while both myeloid and hepatic non‐immune cells were the most prominently modified by IUGR in males.

### 2.4 Females have Advanced Immunological Adaptations to Mitigate IUGR‐Induced Hepatic Injury

**Figure 3:**
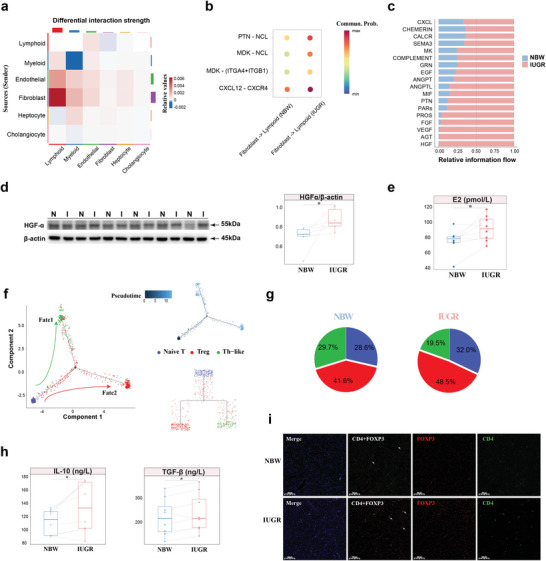
IUGR‐induced hepatic injury alleviated by adaptive transition of T cells in females. a) Differential interaction strength among major cell types between NBW and IUGR females. b) Significant ligand‐receptor interactions in fibroblast and lymphoid cells between NBW and IUGR females. c) Stacked plots displaying significant cell communication signaling pathways. d) Western blot image of HGF expression in the liver of NBW and IUGR females (n = 6). e) The levels of E2 in the serum of NBW and IUGR females using ELISA (n = 8). f,g) The dynamics of CD4+ T cell subclusters and their pseudotime curve in trajectory plots by Monocle2 (f) and the contribution of IUGR and NBW females to each subcluster in CD4+ T cells (g). h, i) The levels of IL‐10 and TGF‐β (h) and immunofluorescence image of CD4 (green), DAPI (blue), and FOXP3 (red) (i) in the liver of NBW and IUGR females using ELISA (n = 8). Scale bar, 50 µm. Two‐sided paired Student's t‐test (d, e, i) (*P < 0.05).

Traditional bulk RNA‐seq lacks information on the interactions of various cell types in the liver during IUGR. A relatively robust interaction between lymphocytes, fibroblasts and endothelial cells was unveiled in IUGR females by CellChat analysis (**Figure** **3**a). The key ligand‐receptor interactions like PTN‐NCL and CXCL2‐CXCR4 had increased activity during IUGR (Figure 3b), suggesting that the activation of fibroblasts might influence the differentiation of lymphoid cellular subsets. There was higher information flow for various growth factors, such as HGF, VEGF, FGF, PTN, and EGF in IUGR females than in NBW females (Figure 3c). We confirmed higher hepatic HGF protein expression levels (Figure 3d) and serum E2 content (Figure 3e) in IUGR females than in NBW females. To further assess the heterogeneity of lymphoid cells in females, Louvain clustering analysis was performed and four major T cell subclusters, five NK cell subclusters and one NKT cluster were identified. Three CD4+ T cell subclusters were identified based on CD4 or CD8 expression, revealing a developmental differentiation trajectory (Figure S7a, Supporting Information). The selected T cell function‐associated genes were compared among CD4+ T cell subclusters, such as those with high expression of CCR7 and IL7R as naïve T cells, FOXP3 and CTLA4 as Treg T cells, and other cells as Th‐like cells (Figure S7b, Supporting Information), respectively. Monocle trajectory analysis of CD4+ T cells revealed a differentiation trajectory originating from naïve T cells and gradually changing to Treg T cells with Th‐like cells branching out (Figure 3f). To evaluate the precision of the constructed trajectory, the expression levels of genes governing the differentiation process of T cells in two fates were observed over pseudotime (Figure S7c, Supporting Information). CCR7, IL7R, and LEF1 are highly expressed during T cell quiescence.^[^ ^24^ ^]^ CCL5 and SH2D1A were highly expressed during T cell activation. HOPX and PDK1 regulate the differentiation of Th‐like cells (Fate1).^[^ ^25^ ^]^ FOXP3 and CTLA4 regulate the differentiation of Treg cells (Fate2).^[^ ^26^ ^]^ The branching heatmap displayed genes with notable dynamic changes between the two fates (Figure S7d, Supporting Information). Th‐like cells (fate1) were linked to regulatory cell activation, whereas Treg cells (fate2) were linked to the downregulation of immune system processes according to GO analysis (Table S7, Supporting Information).

Of particular interest, a higher percentage of Treg cells was detected in IUGR females (48.5% in the IUGR group versus 41.6% in the NBW group) (Figure 3g). This finding was verified by elevated levels of IL‐10 and TGF‐β and a greater positive cell count in IUGR females through enzyme‐linked immunosorbent assay (ELISA) and CD4+FOXP3+ co‐staining (Figure 3h,i). Treg T cells are involved in maintaining immune homeostasis and immune tolerance.^[^ ^27^ ^]^ The increased presence of Treg T cells in IUGR females suggested that liver tissue might transition toward immune tolerance to mitigate hepatic injury caused by IUGR‐induced inflammation one week postnatally.

### 2.5 Males Recruit more Kupffer Cells and Trigger Inflammation by PRDM1 to Worsen IUGR‐Induced Hepatic Injury

**Figure 4:**
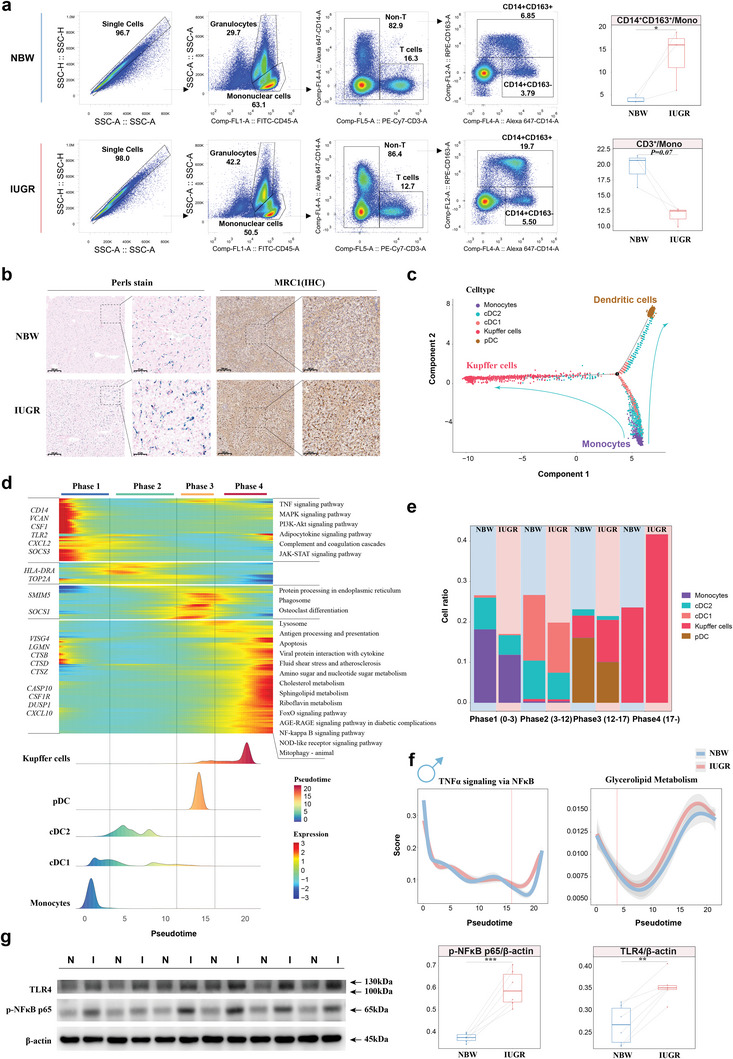
IUGR‐induced hepatic injury worse in males due to the inflammatory transition of Kupffer cells. a) Proportions of T cells and macrophages using flow cytometry between IUGR and NBW males (n = 3). b) Perls stain and immunohistochemistry image of MRC1 showing the distribution of macrophages in males. c) Trajectory plots showing the dynamics of myeloid cells by Monocle2. d) Heatmap revealing the dynamic changes in gene expression during the differentiation process, including four phases and KEGG analysis among different phases along with the pseudotime. e) Stacked histogram indicating the proportion of myeloid cells of each phase between IUGR and NBW males. f) Diagram of the expression scores for TNFα signaling via NFκB and glycerolipid metabolism. g) Western blot images of TLR4 and p‐NFκB p65 expression in the liver of NBW and IUGR males (n = 6). Two‐sided paired Student's t‐test (a, g) (***P < 0.001; **P < 0.01; *P < 0.05). Individual P‐values are denoted above the comparison lines.

To verify the observed decrease in T cell counts and the increase in Kupffer cell counts in IUGR males through scRNA‐seq, flow cytometry was employed to examine the percentages of CD45+CD3E+ and CD45+CD14+CD163+ cells, which were used for labeling T cells and Kupffer cells, respectively (**Figure** **4**a). The observed patterns of both parameters statistically matched the scRNA‐seq data. CellPhoneDB analysis was used to explore the cell‐cell communication network in male liver tissue (Table S8, Supporting Information). A dominant relationship was found between myeloid cells and non‐immune cells, with diminished intercellular signals in IUGR males (Figure S8a,b, Supporting Information). Significant ligand‐receptor (L‐R) pairs, such as PTPRC and MRC1, were activated in IUGR males (Figure S8c, Supporting Information). MRC1 has previously been reported to play a critical role in myeloid plasticity,^[^ ^28^ ^]^ as high expression of MRC1 suggested that IUGR‐induced hepatic injury resulted in the recruitment of more Kupffer cells in males. Perls’ Prussian blue staining and immunohistochemistry (IHC) confirmed that there were more Kupffer cells and stronger MRC1 expression signals in IUGR males than in NBW males (Figure 4b). Dynamic immune states and cell transitions in myeloid cells were explored by inferring state trajectories. As previously described,^[^ ^29^ ^]^ a pseudotime analysis showed that monocytes were located at the beginning of the trajectory path, while DCs and Kupffer cells occupied one of two terminal states (Figure 4c; Figure S8d, Supporting Information). By integrating functional enrichment and trajectory phases (Table S9, Supporting Information), a transition was identified starting with the activation of monocytes via the JAK‐STAT signaling pathway and adipocytokine, progressing through an intermediate proliferative state characterized by cDCs, followed by a phagosomal state featuring pDCs and some Kupffer cells, and culminating in a metabolic disorder and inflammatory state characterized by Kupffer cells (Figure 4d). Kupffer cells predominated in the end‐state, and their fraction was much higher in IUGR males than in NBW males (Figure 4e), indicating that the activation of Kupffer cells and recruitment occurred in some way. We observed that IUGR males had higher scores of TNFα signaling via NFκB at terminal phase and high scores of glycerolipid metabolism after the period of monocytes by AddModule Score (Figure 4f), but this effect was not observed in female myeloid cells (Figure S8e, Supporting Information). We confirmed that IUGR males were in an inflammatory state with higher protein expression levels of TLR4 and phospho‐NFκB p65 than NBW males (Figure 4g). Our multifaceted results indicated that Kupffer cells were significantly affected in IUGR males.

Non‐negative matrix factorization (NNMF) was performed to cluster cells to explore the heterogeneity within Kupffer cells (Table S10, Supporting Information). Kupffer cells were clustered into three different subtypes, and exclusive factors for functional enrichment in each subtype were determined (Figure S9a, Supporting Information). The inflammatory subtype was enriched in the TNF signaling pathway and highly expressed NFKBIZ, KLF6, TNF, and DUSP1, resembling M1 macrophages;^[^ ^30^ ^]^ the non‐inflammatory subtype was enriched in oxidative phosphorylation and highly expressed RACK1, ATP5PF, RPL36AL, and TMSB10, resembling M2 macrophages;^[^ ^30^ ^]^ and proliferation was characterized by MKI67 and TOP2A (Figure S9a,b, Supporting Information). The scores of the pro‐ and anti‐inflammatory gene sets were used to validate the accuracy of classifying subtypes (Figure S9c, Supporting Information). Compared with NBW males, IUGR males had a higher percentage of inflammatory subtypes (45.7% in the IUGR group vs 36.8% in the NBW group). A potential transformation from the non‐inflammatory subtype to the inflammatory subtype was suggested by mapping them along a pseudotemporal trajectory and interrogating their directionality via spliced and unspliced mRNA ratios (RNA velocity) (Figure S9d, Supporting Information). Several genes were highlighted in the pseudotemporal dynamic heatmap (Figure S9e, Supporting Information), including PSAP mediating the activation of macrophages,^[^ ^31^ ^]^ and SESN3 regulating the levels of reactive oxygen species.^[^ ^32^ ^]^ Gene set variation analysis (GSVA) (Figure S9f, Supporting Information) and DEGs analysis (Figure S9g, Supporting Information) revealed distinct differences in the inflammatory subtype between NBW and IUGR males. The inflammatory subtype in NBW males showed a greater score for the reactive oxygen species pathway, resembling classic M1 macrophage capacity to produce ROS.^[^ ^33^ ^]^ In contrast, IUGR individuals exhibited high scores of hypoxia and lipid homeostasis. Accordingly, to explore the existence of different regulatory models for this process, pySCENIC was employed to gain mechanistic insight into transcriptional regulation of subtypes. The top 5 specific transcriptional factors were displayed for each cell type and mapped to the UMAP plots (Figure S9h, Supporting Information). By comparing the transcriptional regulation of the inflammatory subtype between NBW and IUGR individuals, higher regulon activity scores for PRDM1 were observed in IUGR males (Figure S9i,j; Table S11, Supporting Information). PRDM1 was associated with studies on macrophages in the liver using the SEEK datasets (Figure S9k, Supporting Information). In brief, IUGR‐induced hepatic injury in males resulted in the recruitment of more Kupffer cells and the activation of PRDM1 to induce the inflammatory subtype, which might be related to a hypoxic environment or altered lipid metabolism.

### 2.6 Inflammatory Environment Disrupts Communication Networks Among Subpopulations in Hepatic Non‐Immune Cells in IUGR Males

Analyzing non‐immune cells, we identified 14 clusters using Louvain clustering. These clusters included seven distinct subtypes of endothelial cells (Endo), four subtypes of fibroblasts, and two subtypes of hepatocytes (Hep) and cholangiocytes. Each subtype had unique signature genes (Figure S10a,b; Table S12, Supporting Information). A comparison of subgroup proportions revealed variations in the makeup of hepatic epithelium cells between IUGR males and NBW individuals (Figure S10c, Supporting Information). The inextricable link between endothelial cells and liver regeneration has been of great interest,^[^ ^34^ ^]^ which prompted us to perform GO analysis on the endothelial subtypes based on enriched markers to comprehensively unravel their heterogeneity and function (Table S13, Supporting Information). The liver vascular endothelium comprises liver sinusoidal endothelial cells (LESCs) and the endothelium of blood vessels. Endo1, Endo2, and Endo3 displayed functional similarities to the LESCs, while Endo4, Endo5, and Endo6 were related to the endothelium of blood vessels. According to previous studies,^[^ ^35^ ^]^ we identified Endo1 and Endo2 as periportal LSECs (OIT3, FCN2, HGF); Endo3 as central venous LSECs, enriched in immune pathways; Endo4 as vein ECs (CD34, PECAM1); Endo5 as capillary ECs (RGCC); Endo6 as lymphatic ECs (NRP2), and Endo7 as endothelium‐derived stromal cells (APOLD1, CD9, MMP2), exhibiting cytoplasmic translation and extracellular matrix organization (Figure S10d, Supporting Information).

**Figure 5:**
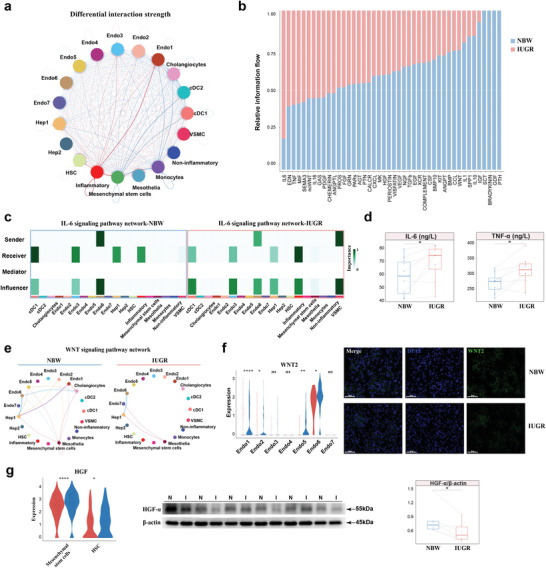
Hepatic non‐immune cells and their communication impaired by inflammation in IUGR males. a) Network chart showing differential interaction strength of non‐immune cells and myeloid cells between IUGR and NBW males. Blue: NBW‐upregulated interaction. Red: IUGR‐upregulated interaction. b) Stacked plots displaying significant cell communication signaling pathways. c) Heatmap plots showing the role of cell types involved in the IL‐6 signaling pathway between IUGR and NBW males. d) The levels of IL‐6 and TNF‐α in the liver of NBW and IUGR females using ELISA (n = 8). e) Network charts showing the complexity of WNT signaling pathway. f) Expression levels of WNT2 in scRNA‐seq and immunofluorescence image of WNT2 (green) and DAPI (blue). g) Expression levels of HGF in scRNA‐seq and western blot image of HGF expression in liver of NBW and IUGR males (n = 6). Scale bar, 50 µm. Two‐sided paired Student's t‐test (d, g) and unpaired Wilcoxon rank‐sum test (f, g) (****P < 0.0001; **P < 0.01; *P < 0.05; ns *P* > 0.05).

To elucidate the potential cellular communication involved in IUGR‐induced hepatic injury in males, the potential theoretical cell communication between myeloid and non‐immune populations was calculated by CellChat (Table S8, Supporting Information). A connection graph visualized that IUGR individuals had significantly upregulated strength of signaling involved in the inflammatory subtype, but downregulated communication associated with epithelial, endothelial, and mesenchymal cells (**Figure** **5**a). A total of 44 pathways showed notable differences in signal strength for IUGR males (Figure 5b). The signaling of PTH, GDF, BRADYKININ, and SCT was entirely eliminated in IUGR males. IUGRs also led to low activity in most information flows, including the production of anti‐inflammatory factors (IL‐10, CSF), factors regulating growth and development (IGF, BMP, TGF‐β), and factors promoting liver regeneration (WNT, HGF, EGF). However, IUGR activated pro‐inflammatory signalings (IL‐6, TNF‐α, MIF). Global communication pattern recognition analysis showed that different cell types play specific roles (e.g., sender, receiver, mediator, and influencer) in intercellular information exchange. Hep1 and cholangiocytes functioned in GDF and SCT signaling, respectively (Figure S11a,b, Supporting Information). The majority of cell types were influenced by IL‐6 signaling due to IUGR (Figure 5c). The significant increase in TNF‐α and IL‐6 levels was confirmed in IUGR males compared with NBW males using ELISA (Figure 5d). IUGR caused a simpler cellular communication network for WNT (Figure 5e) and TGF‐β (Figure S11c, Supporting Information), and lacked the expression of relevant ligand‐receptor pairs (Figure S11d–f, Supporting Information). The weak intensity of WNT2 and HGF was confirmed in males suffering from IUGR using immunofluorescence (IF) or western‐blotting (WB) (Figure 5f,g). Durable liver regeneration is dependent on the expression of WNT2 and HGF.^[^ ^36^ ^]^ Our data showed that in males, IUGR‐induced inflammation caused a marked perturbation of hepatic non‐immune cell communication networks.

### 2.7 TG Deposition Caused by Liver Dysfunction as Altered Metabolic Outcomes are Requisite Factors for Exacerbating Inflammation

**Figure 6:**
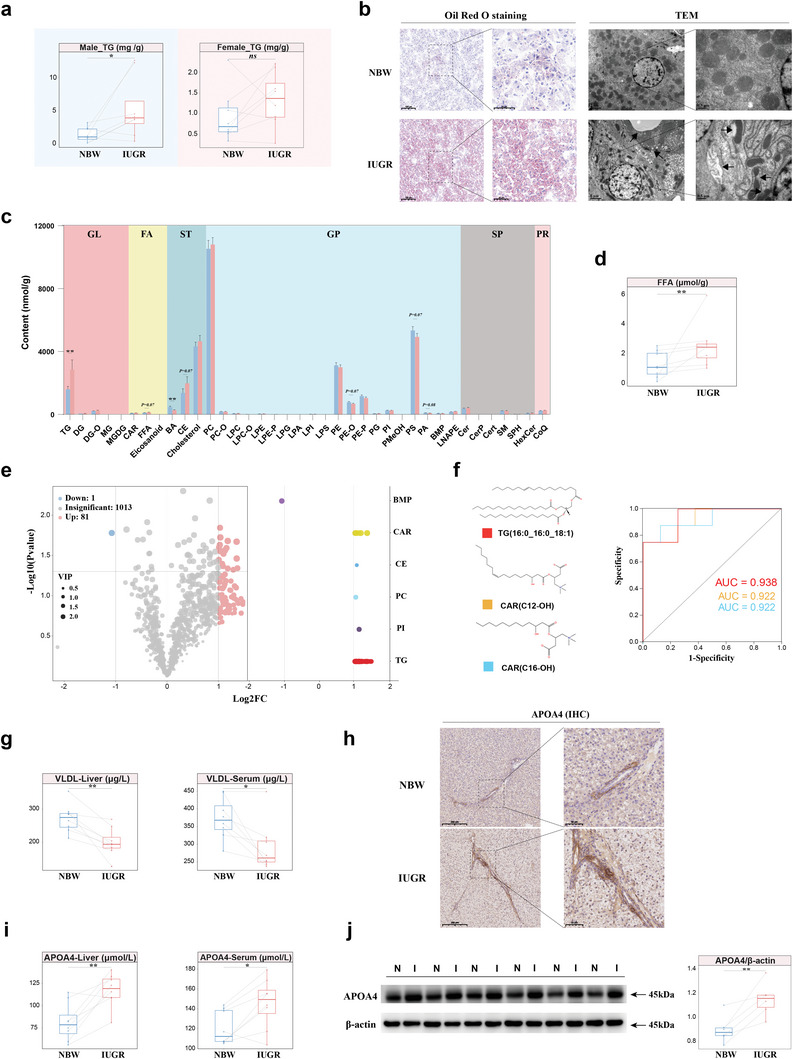
Profiles of altered lipid metabolism induced by hepatocellular injury in IUGR males. a) Comparison of TG levels in the liver of NBW and IUGR piglets of both genders (n = 8). b) Histopathology of liver assessed by Oil Red O staining and TEM between NBW and IUGR males. Right: Magnified images of indicated dotted box areas. c) Quantity of different classes of lipids by UPLC‐MS/MS. d) The concentration of FFA in the liver of NBW and IUGR males (n = 8). e) Volcano plot showing differential lipids and their classes between IUGR and NBW males. f) The chemical structure and ROC curve of CAR(C12‐OH), CAR(C16‐OH) and TG(16:0_16:0_18:1). g) The concentration of VLDL in the liver and serum of NBW and IUGR males (n = 8). h) Immunohistochemistry image of APOA4 in the liver of NBW and IUGR males. i, j) The levels of APOA4 in the liver and serum using ELISA (n = 8) (i), and western blot image of APOA4 expression (j) of NBW and IUGR males (n = 6). Scale bar, 200 µm and 100 µm or 2 µm and 0.5 µm, 200 µm and 50 µm (c). Two‐sided paired Student's t‐test (a, d, g, i, j) and unpaired Wilcoxon rank‐sum test (c) (**P < 0.01; *P < 0.05, ns *P* > 0.05). H&E, hematoxylin‐eosin staining; PAS, periodic acid‐schiff staining; TEM, transmission electron microscope. VLDL, very low density lipoprotein. CAR, carnitine; TG, triglyceride; GL, glycerolipids; FA, fatty acids; ST, sterol lipids; GP, glycerophospholipids; SP, sphingolipids; PR, prenol lipids.

Compared with IUGR females, IUGR males exhibited more pronounced TG accumulation in the liver (**Figure** **6**a). Further analyses of hepatocellular subtypes revealed that Hep2 had significantly fewer mRNA features and counts than Hep1 (Figure S12a, Supporting Information). Thus, GO analysis was performed to examine functional changes in Hep1. IUGR males were mainly enriched in cytoplasmic translation and ATP metabolic process, reflecting hepatocellular damage.^[^ ^37^ ^]^ A series of hepatic pathological sections confirmed that IUGR males exhibited more pronounced hepatic injury and altered hepatic function than IUGR females (Figure S12b,c, Supporting Information). Hematoxylin‐eosin staining (H&E) and periodic acid‐schiff staining (PAS) showed marked inflammatory lymphocytic infiltration and apparent vacuoles, along with the accumulation of glycogen in IUGR males (Figure S12b, Supporting Information). Oil Red O staining and transmission electron microscopy (TEM) demonstrated the accumulation of lipids and loss of cytoplasmic material, an increase in lipid droplets, degeneration of mitochondria, and endoplasmic reticulum in IUGR males (Figure 6b).

Lipid metabolomics was used to quantify the pattern of lipid changes in the livers of IUGR males. After rigorous quality control (Figure S13a,b, Supporting Information), a total of 1095 lipids spanning 38 lipid subclasses from 6 major classes were identified in the samples (Figure S13c; Table S14, Supporting Information). Both dendrogram cluster analysis and orthogonal projections to latent structures discriminant analysis (OPLS‐DA) depicted distinct patterns of lipids between NBW and IUGR males (Figure S13d, Supporting Information). Sorting the lipids by content revealed that the majority of lipids had higher contents in IUGR males than in NBW males (Figure S13e, Supporting Information). The contents of each lipid subclass between NBW and IUGR males are displayed (Figure 6c), and IUGR males had high levels of free fatty acids (FFA) (Figure 6d). The volcano plot highlighted 82 differential lipids (81 up‐ and 1 down‐regulated) between IUGR and NBW males, mainly belonging to the TG and carnitine (CAR) classes (Figure 6e). Moreover, sorting the lipids by fold changes identified the top 10 up‐ and down‐regulated lipids in IUGR and NBW males (Figure S13f, Supporting Information). The z‐score plot depicts the disordered profile of lipids due to IUGR (Figure S13g, Supporting Information). Altered lipids in IUGR males had enriched functional pathways related to insulin resistance and glycerolipid metabolism, according to the KEGG database (Figure S13h, Supporting Information). BMP (20:4_22:6), a redox‐sensitive enhancer of lipolysis,^[^ ^38^ ^]^ was the only downregulated lipid in IUGR males. The upregulation of CARs, which are involved in fatty acid transport,^[^ ^39^ ^]^ indicated a dysfunctional β‐oxidation process of fatty acids in the mitochondria of IUGR males. Closely connected to that finding, we observed a deposition of multiple TGs due to IUGR, with a positive correlation in content among them (Figure S13i, Supporting Information). The length of carbohydrate chains or saturation largely determines the function of metabolites.^[^ ^40^ ^]^ TGs with chains of 48 and 50 carbon atoms, or TGs with 1 to 5 double bonds, composed the majority of differential TGs (Figure S13j, Supporting Information). Finally, CAR(C12‐OH), CAR(C16‐OH), and TG(16:0_16:0_18:1) were strongly associated with IUGR by ROC curves analysis (Figure 6f).

ELISA revealed reduced levels of very low‐density lipoprotein (VLDL) in hepatic tissue and serum of IUGR males (Figure 6g). As VLDL plays a crucial role in hepatic TG transport and clearance,^[^ ^41^ ^]^ the diminished secretion of VLDL in IUGR males might act as a pivotal factor contributing to the accumulation of TG. We noticed variations in the expression levels of genes related to the apolipoprotein family in both Hep1 and in the RNA‐seq data (Figure S14a,b, Supporting Information). Surprisingly, APOA4 consistently and robustly upregulated in IUGR males across different sequencing strategies, whereas this situation was not present in IUGR females (Figure S14c, Supporting Information). Increased APOA4‐positive hepatocytes were observed around the portal triad due to IUGR (Figure 6h). APOA4 contents were higher in both serum and liver due to IUGR by ELISA (Figure 6i). Elevated APOA4 mRNA and protein expression levels were also validated in the liver of IUGR males (Figure 6j; Figure S14d, Supporting Information).

**Figure 7:**
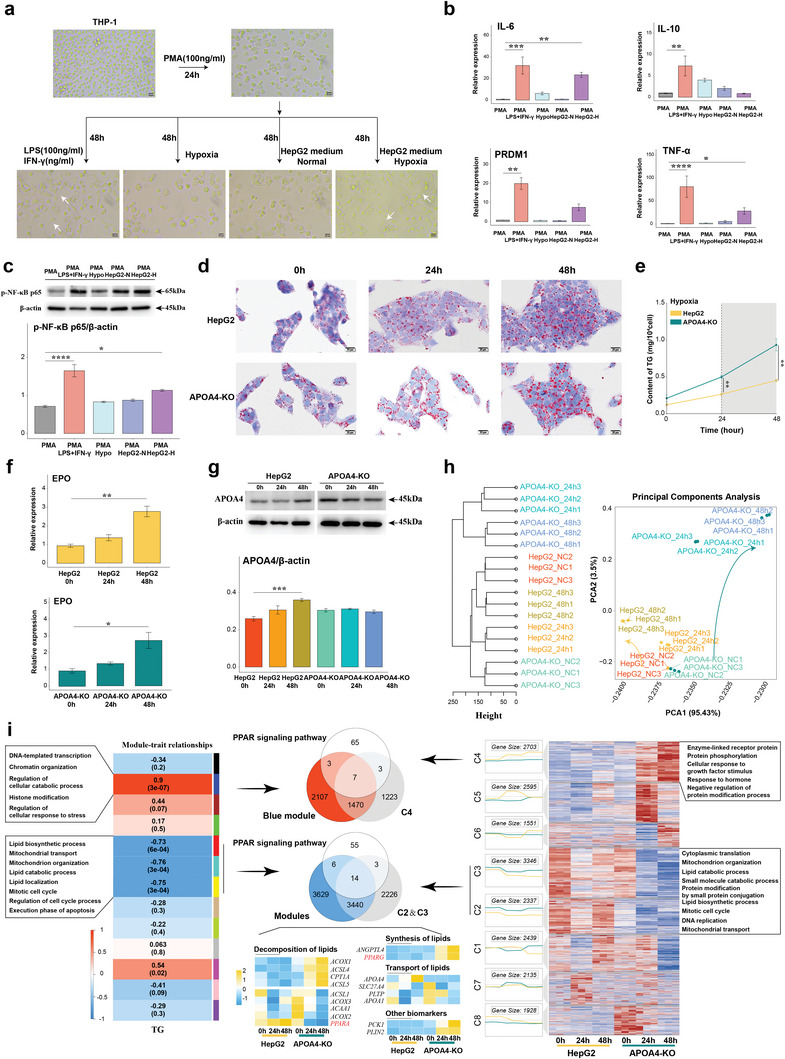
Hepatocellular injury promotes inflammation and upregulation of APOA4, which mitigates TG deposition in hepatocytes under hypoxia. a–c) Morphology images of macrophages (a) and mRNA expression of IL‐6, IL‐10, PRDM1, and TNF‐α (b), and Western blot image of p‐NFκB p65 levels (c) under LPS and IFN‐γ, hypoxia, normal HepG2 medium, and HepG2 medium with hypoxia condition. d) Histopathology of HepG2 in normal or with APOA4‐KO under hypoxia in Oil Red O staining. e) Alterations in TG levels were observed within the HepG2 and APOA4‐KO during hypoxia. f,g) The level of EPO mRNA (f) and APOA4 protein (g) within the HepG2 and APOA4‐KO during hypoxia. h) PCA analysis of transcriptional profiles within the HepG2 and APOA4‐KO during hypoxia. i) The integrated and detected intersection of gene clustering associated with TG levels or temporal variations. Scale bar, 20 µm (a, d). Data presented as mean±SD (n = 3). Ordinary one‐way ANOVA with Tukey's multiple comparisons test (b, c, f, g) and unpaired Wilcoxon rank‐sum test (e) (****P < 0.0001; ***P < 0.001; **P < 0.01; *P < 0.05).

To explore the close relationship between hypoxia‐induced liver damage and inflammation, macrophages were induced by PMA in THP‐1 cells and subjected to four interventions: a) LPS and IFN‐γ treatment as “positive” controls, b) 1% oxygen conditions, c) medium of HepG2 cell line cultured under hypoxia conditions (1% oxygen), and d) medium of HepG2 cell line cultured under normal conditions (21% oxygen) (**Figure** **7**a). Cells exhibited a pike‐shaped morphology with extended pseudopods, indicating differentiation toward the M1 phenotype. The mRNA levels of IL‐6, TNF‐α, PRDM1, and IL‐10 were significantly up‐regulated in M1 macrophages as positive controls (Figure 7b). Interference with the medium for the HepG2 cell line under hypoxic conditions (c) increased mRNA levels of inflammatory factors such as IL‐6 and TNF‐α, and transcription factor, like PRDM1, while the direct hypoxia culture treatment resulted in an increase in mRNA levels of IL‐10. Protein levels of phospho‐NFκB p65 in macrophages exposed to the hypoxia‐based culture medium of HepG2 cell line also appeared to be elevated (Figure 7c). Taken together, our findings suggest that metabolic disturbances derived from hypoxia‐induced liver damage promote macrophage differentiation toward the M1 phenotype.

### 2.8 APOA4 Regulates TG Deposition by Mediating the PPAR Signaling Pathway to Alleviate Hypoxia‐Induced Hepatocellular Injury

The significance of APOA4 overexpression, which is confined to hepatocytes, remained somewhat elusive (Figure S14e, Supporting Information). The amino acid sequence of APOA4 was highly homologous (80.11%) between pigs and humans (Figure S14f, Supporting Information).

We employed CRISPR‐Cas9 technology to generate specific knockouts for three exonic regions of APOA4 to better understand the regulatory role of APOA4 in hepatic lipid metabolism during hypoxia‐induced damage. We successfully established an APOA4‐KO HepG2 cell line (Figure S15a,b, Supporting Information). The absence of APOA4 significantly reduced the seeding efficiency of hepatocytes without influencing the cell cycle (Figure S15c, Supporting Information). The results showed a steady increase in intracellular TG content with extended hypoxia duration, and the absence of APOA4 accelerated TG deposition (Figure 7d,e). EPO mRNA levels were monitored to assess the efficacy of the hypoxia model (Figure 7f), and a noticeable increase in the APOA4 protein level was confirmed under hypoxia conditions (Figure 7g).

To comprehensively understand the function of APOA4 in the context of hypoxia, we used RNA‐seq to compare mRNA expression profiles of two cell lines at 0, 24, and 48 h after hypoxia treatment (Table S15, Supporting Information). Principal component analysis (PCA) revealed significant differences in gene expression patterns between the APOA4‐KO HepG2 cell line and the normal HepG2 cell line following hypoxia (Figure 7h). With prolonged exposure to hypoxia, an increased number of DEGs were identified, particularly those related to metabolic pathways (Figure S15d, Supporting Information). The absence of APOA4 was found to cause sustained disruptions in the PPAR signaling pathway, as indicated by gene set enrichment analysis (GSEA). By using weighted gene co‐expression network analysis (WGCNA) and gene time series clustering analysis, we successfully identified gene modules that were positively or negatively correlated with TG levels or the duration of hypoxic treatment (Figure 7i). The mRNA expression levels of overlapping genes obtained from the aforementioned modules and the list of genes associated with the PPAR signaling pathway were examined using RT‐qPCR. The results revealed that the absence of APOA4 influenced lipid decomposition (e.g., ACOX1, ACSL4, CPT1A, ACSL5, ACSL1, ACOX3, ACAA1, ACOX2, PPARα), synthesis (e.g., ANGPTL4 and PPARγ) and transport (e.g., APOA4, SCL27A4, PLTP, APOA1), and promoted the expression of PCK1, a key enzyme in gluconeogenesis, and PLIN2, a biomarker of lipid droplets.^[^ ^42^ ^]^ These findings collectively implicate that the pronounced increase in APOA4 expression in IUGR males may represent a potential, protective mechanism against hypoxia. Previous studies have also suggested that enhanced APOA4 expression could alleviate hepatic lipid disorder.^[^ ^43^ ^]^

### 2.9 Modulation of the PPAR Signaling Pathway Repairs IUGR‐Induced Liver Damage to Alleviate APOA4 Expression in Males

**Figure 8:**
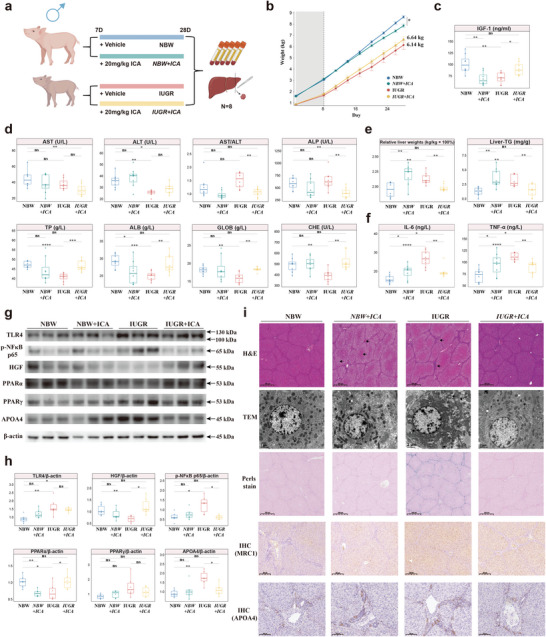
Hepatic injury in IUGR males orally inoculated with PPARα agonists. a) Schematic diagram of experimental design (n = 8). b) Line plots over time depicting the temporal change in growth performance across groups. c) IGF‐1 levels in the serum. d) Serum parameters related to liver function. e) Relative liver weight and hepatic TG content. f) Levels of IL‐6 and TNF‐α in the liver. g, h) Western blot image of TLR4, p‐NFκB p65, HGF, PPARα, PPARγ and APOA4 expression with statistical tests (n = 6). i) Histopathology of liver assessed by H&E, TEM, Perls stain and immunohistochemistry image of MRC1 and APOA4 in the liver. Scale bar, 500 µm, 2 µm (g). Ordinary one‐way ANOVA with Tukey's multiple comparisons test (b, c, d, e, f) (****P < 0.0001; ***P < 0.001; **P < 0.01; *P < 0.05).

Icariin (ICA) functions as an agonist of PPARα, effectively modulating the PPAR signaling pathway to exert a lipid‐lowering effect.^[^ ^44^ ^]^ Male IUGR piglets were orally administered ICA (20 mg kg^−1^ BW) daily from 7d to 28d, and liver and serum samples were collected after euthanasia (**Figure** **8**a). IUGR males treated with ICA exhibited improved growth performance (Figure 8b) and increased IGF‐1 levels in serum (Figure 8c). Similarly, various parameters of liver function in serum tended to normalize, approaching the levels observed in NBW individuals (Figure 8d). At the same time, IUGR males treated with ICA displayed a significant reduction in relative liver weight and liver TG content compared with IUGR males (Figure 8e). Amelioration of inflammation in IUGR males treated with ICA was confirmed by a decrease in the levels of inflammatory factors, like IL‐6 and TNF‐α, in comparison to those in IUGR males (Figure 8f). Protein expression of PPARα and HGF was increased, and the protein expression of phospho‐NFκB p65 was decreased in IUGR males treated with ICA compared with IUGR males (Figure 8g,h). Interestingly, a significant downregulation of APOA4 and a decreased number of APOA4‐positive hepatocytes around the portal triad were observed in IUGR males treated with ICA compared with IUGR males (Figure 8g–i). Validation by a series of histological staining showed the efficacy of ICA in mitigating IUGR‐induced liver damage, including a reduction in vacuolization, attenuation of immune infiltration, restoration of mitochondrial morphology, reduction of lipid droplets, and diminished recruitment of Kupffer cells (Figure 8i; Figure S16a, Supporting Information). The above findings indicate the intricate connection between the expression of APOA4 during IUGR and the PPAR signaling pathway, highlighting the considerable potential of APOA4 as both a therapeutic target and a biomarker for IUGR‐induced liver damage.

## 3 Discussion

IUGR in humans is considered a major worldwide public health concern, while its primary prevention still has an unacceptably slow pace.^[^ ^45^ ^]^ The occurrence of IUGR has a wide‐reaching impact on virtually all organs and systems,^[^ ^46^ ^]^ prompting the exploration of adaptive mechanisms that enable affected individuals to endure IUGR. With these facts in mind, our investigation focused on uncovering a sex‐related aspect of liver function. Based on an unbiased and comprehensive cellular landscape using scRNA‐seq, distinct patterns in the liver of males and females during the early postnatal period were observed in response to IUGR (Figure S18, Supporting Information). In IUGR females, fibroblasts seemed to be activated by E2 to drive the differentiation of Treg cells, which then alleviated inflammation and promoted liver regeneration. In contrast, more severe liver damage occurred in IUGR males, and a parallel association existed between altered metabolism and inflammation following hypoxia‐induced hepatocellular injury. In IUGR males, the inflammatory environment disrupted non‐immune cellular communication networks, weakening liver regeneration signals that persisted into adulthood. Our study corroborates the assumption that it is necessary to analyze IUGR through a sex‐specific lens.^[^ ^9a^ ^]^ Finally, APOA4 had been identified as a new biomarker and therapeutic target for IUGR in males, because it mitigates TG deposition in the context of hypoxia‐induced hepatocellular damage through the PPAR signaling pathway. PPARα activation with an agonist could mitigate hepatic damage and simultaneously restore over‐expressed APOA4 to normal levels in male IUGR individuals.

Compared to mice, pigs possess a more human‐like immune system and other physiological similarities, especially during the neonatal stage,^[^ ^10^ ^]^ which make them ideal subjects for organ transplantation research. Recent scientific studies have harnessed the power of scRNA‐seq to identify the degree of homogeneity of cellular elements between pigs and humans.^[^ ^47^ ^]^ To further advance human medicine, comprehensive multi‐tissue/organ maps of pigs as model systems for translational approaches are required.^[^ ^35^, ^48^ ^]^ Our data support that the liver has conserved cellular transcriptional architectures and programs between pigs and humans, particularly in macrophages, as shown for the conserved hepatic macrophage niches across species.^[^ ^49^ ^]^ This observation sets the stage for a translational clinical application of our study in the field of hepatic pathology during IUGR in humans. Several research studies have investigated how the liver is affected by naturally occurring IUGR in pigs over time.^[^ ^9^, ^11^ ^]^ On the other hand, males seem to be more susceptible to IUGR‐induced homeostatic disruptions.^[^ ^9a^ ^]^ Liver function tests revealed altered serum indicators for IUGR male piglets, such as AST, ALT, ALB, TP, as well as unusual levels of ALP and CHE activity. The reduced activity of CHE, a sensitive and specific diagnostic marker for liver diseases,^[^ ^50^ ^]^ suggested that liver dysfunction was involved in IUGR in males. Our piglet model can effectively simulate infants with hepatic changes to study alterations in males specifically due to IUGR.

As widely acknowledged, the resilient and immune‐rich environment of the liver supports regional and systemic homeostasis, enabling hepatic regeneration.^[^ ^51^ ^]^ However, the delicate equilibrium of liver homeostasis is perturbed by the process of intrauterine programming caused by an unfavorable intrauterine environment.^[^ ^52^ ^]^ It is well‐established that IUGR leads to intrauterine hypoxia, a fact validated by data showing an enrichment of the HIF‐1 signaling pathway and highlighting the expression of EPO and GAPDH during IUGR. Higher numbers of erythroid cells were captured in individuals suffering from IUGR, confirming that intrauterine hypoxia caused increased, compensatory synthesis of erythrocytes.^[^ ^53^ ^]^ Of particular interest was the observation that IUGR females appeared to be less severely affected by inflammation and altered lipid metabolism in comparison to their male counterparts. Eventually, IUGR females are accompanied by a higher level of hepatic regeneration signals. In humans, women may be more sensitive and adapt to the intrauterine environment, as previously theorized.^[^ ^54^ ^]^ Our study found that adaptation to the adverse environment involved the transition of lymphoid cells and monocytes toward an immune‐tolerant phenotype in females suffering from IUGR. Monocytes were activated in females, as evidenced by enrichment of pathways like the cellular response to cytokine stimulus. The activation of monocytes is triggered by cell signaling factors produced by activated T cells.^[^ ^55^ ^]^ In this context, an elevated proportion of Treg cells and increased secretion of related cytokines were also observed in females suffering from IUGR. A previous study has reported that Treg cells possess the capacity to modulate immune responses and mitigate tissue damage resulting from autoreactive or excessive inflammatory immune responses.^[^ ^55^ ^]^Therefore, females confronted with IUGR did not exhibit altered transcriptional profiles of non‐immune cells, scores of inflammation, and lipid metabolism following the peusedotime analysis of myeloid cells. The interaction between fibroblasts and T cells was confirmed to be mediated by the ligand‐receptor pair of CXCL12‐CXCR4 and to trigger the differentiation of Treg cells, as CXCL12 signaling possessed the ability to stimulate T cell chemotaxis and gene expression via its receptor CXCR4.^[^ ^56^ ^]^ The origin of all events observed in females suffering from IUGR might be attributed to the elevated level of E2. E2 has previously been shown to promote hepatic regeneration and regulate hepatic lipid metabolism.^[^ ^57^ ^]^ However, males were not as advantaged.

It has been postulated that Kupffer cells may play an important role in IUGR‐induced hepatic injury.^[^ ^11b^ ^]^ Thus, the altered hepatic metabolism observed in IUGR, such as elevated levels of TG,^[^ ^58^ ^]^ has garnered ongoing attention. In our study, the widespread recruitment and excessive inflammatory response of Kupffer cells within the hepatic milieu of IUGR males were closely intertwined with the disruptions in hepatic lipid metabolism. Altered profiles of lipids in IUGR males had been demonstrated in Table S14, Supporting Information. Altered abundance and composition of lipids in liver tissue could lead to the clustering of Kupffer cells.^[^ ^59^ ^]^ We quantified the presence of CD14+CD163+ cells and scrutinized the spatial distribution of MRC1 to confirm that recruitment. In line with earlier studies highlighting the elevated MRC1 expression at tissue pathology sites during diseases,^[^ ^60^ ^]^ IUGR males had a more pronounced MRC1 signaling profile. A high frequency of communication signals was observed in Kupffer cells and non‐immune cells, validating the concept of “integrated and intercellular circuits”,^[^ ^61^ ^]^ in which hepatocytes, LSECs, HSCs, and Kupffer cells function collaboratively in an intercellular manner. Liver pathology has been linked to the recruitment of monocyte‐derived macrophages, although their fate remains enigmatic.^[^ ^61^ ^]^ Kupffer cells identified in IUGR males suggested a transition toward both an inflammatory state and a metabolically disruptive state as inferred from integrated trajectory information and key gene sets scoring within the milieu of myeloid cells. Kupffer cells and their polarized phenotypes are instrumental in IUGR males for multiple reasons. Both M1 and M2 gene signatures are co‐expressed within macrophage subsets based on extensive scRNA‐seq datasets.^[^ ^62^ ^]^ In contrast, our study used NNMF to identify inflammatory and non‐inflammatory subgroups. Our approach, based on NNMF, enhances clustering performance by facilitating the recovery of biologically meaningful gene‐gene correlations.^[^ ^63^ ^]^ It is likely that the higher inflammation in individuals with IUGR was mediated by the activation of PRDM1. Additionally, Kupffer cells in the terminal state of IUGR showed higher scores of the NF‐κB signaling pathway, in line with the notion that the NF‐κB signaling pathway may be involved in PRDM1 to induce M1 polarization.^[^ ^64^ ^]^ Pro‐inflammatory cytokines like IL‐6 and TNF‐α are drivers of liver regeneration,^[^ ^65^ ^]^ while persistent activation of the IL‐6 signaling pathway is detrimental to the liver.^[^ ^66^ ^]^ The inflammatory environment in IUGR males led to the sustained release of IL‐6 and TNF‐α, which disrupted systemic homeostasis by interfering with the communication networks between Kupffer cells and non‐immune cells, resulting in impaired secretion of IGF‐1 and liver regeneration signals, including WNT signaling, HGF, EGF, and TGF‐β. These repercussions persisted into adulthood, as evidenced by diminished cell cycle‐ and Hippo signaling pathway activity, which are both critical for liver cell proliferation, development, and regeneration.^[^ ^67^ ^]^

We observed altered mitochondrial morphology and hepatocellular damage with TG deposition in IUGR males. A strong association was identified between TG levels (16:0_16:0_18:1) and the occurrence of IUGR in males. The altered content of CARs during IUGR is a marker of mitochondrial dysfunction.^[^ ^39^ ^]^ Recently, it was shown that fatty acids are broken down more easily by oxidation in males than in females,^[^ ^68^ ^]^ similar to other sex‐specific alterations in the levels of TG in both liver and serum among individuals affected by IUGR. Another major factor contributing to the deposition of TG is the diminished capacity of hepatocytes to transport lipids into the bloodstream.^[^ ^69^ ^]^ Consistently, our study found lower levels of VLDL in both the liver and serum of IUGR males. Due to increased energy requirements, IUGR males supposedly took up more fatty acids. However, mitochondrial dysfunction impaired FFA oxidation and malfunctioning hepatocytes had decreased transport capacity. This ultimately led to higher TG synthesis rates and increased relative liver weights. Altered lipids promote adverse interactions between hepatocytes and Kupffer cells.^[^ ^70^ ^]^ We confirmed that hypoxia‐induced hepatic damage stimulates macrophage inflammation, accompanied by upregulation of PRDM1 expression and activation of the NF‐κB signaling pathway, through hypoxia‐stimulated, IUGR‐induced hepatic injury in co‐culture cell experiments. Our findings might explain why IUGR males have a disadvantage in survival compared with that of female offspring.^[^ ^8^ ^]^ Thus, PRDM1 holds significant potential as an early therapeutic target for shaping macrophage function and polarization to offset hepatic injury in IUGR males.

Which adaptations enable IUGR males to endure such unfortunate conditions? APOA4, a member of the apolipoprotein family, displayed increased expression in IUGR males. APOA4 has been linked to the regulation of lipid metabolism and possesses anti‐inflammatory properties. Hepatic APOA4 expression was initially considered specific to rodents, but it is also present in human liver and liver cell lines.^[^ ^71^ ^]^ The expression of APOA4 was confirmed in porcine hepatocytes by scRNA‐seq. The high homology in the amino acid sequences of APOA4 between pigs and humans suggests that the pig is an even better model than a knockout mouse for studying the function of APOA4 in liver tissue. There are limited studies available on APOA4 in liver cell lines, and the function of APOA4 during hypoxia remains unknown. Our study showed that there was a time‐dependent upregulation of both APOA4 and TG in hepatocytes following hypoxia. Hepatocytes lacking APOA4 were more susceptible to hypoxic conditions, which led to exacerbated TG accumulation. Similar to the findings in APOA4‐KO rats, there was no significant change in hepatic TG content, but fasting‐induced hepatic steatosis was more severe.^[^ ^72^ ^]^ Unlike in APOA4‐KO rats, the expression of genes related to global and overview maps in metabolism significantly changed in hepatocytes in the absence of APOA4. Undoubtedly, APOA4 regulated TG deposition through the PPAR signaling pathway, affecting various aspects, including decomposition, synthesis, and transport. Additionally, activation of PPARα was sufficient to treat IUGR‐induced TG deposition and inflammation in male livers, accompanied by a reduction in APOA4 over‐expression. The upregulation of APOA4 served as a protective mechanism in IUGR‐induced liver injury, indicating its potential as a novel biomarker for assessing the severity of liver damage in IUGR males.

Our study has several limitations that require consideration. Firstly, the limitation of female single‐cell sequencing samples was due to the lower prevalence of IUGR in females during the sampling period. To mitigate batch effects, we opted for contemporaneous sample selection. However, to validate findings from “omics” data, multiple female IUGRs were included. Secondly, the response patterns of interactions between lymphoid, myeloid, and non‐immune cells affected by IUGR differed between males and females. Unfortunately, our study also lacked a spatial dimension to validate paracrine or intercellular contacts. Thirdly, the intricate mechanisms governing survival under more severe liver injury in males suffering from IUGR remain elusive. In future investigations, it will be necessary to explore the positive and negative feedback regulatory mechanisms involved in the aberrant expression of APOA4, which represents an organismal protective response, and the regulators of the PPAR signaling pathway during IUGR‐induced liver injury.

In conclusion, sex‐related responses of the liver experiencing IUGR one week after birth were further deciphered via scRNA‐seq. Unlike in IUGR females, who could adaptively regulate inflammation to repair liver injury, early interventions are crucial for IUGR males to mitigate the fragility and sensitivity of the liver in adulthood. These interventions should focus on enhancing lipid metabolism, which is a prerequisite for mitigating the excessive inflammation that leads to more severe liver injury. Our results may provide a useful reference for the development of sex‐specific strategies for early interventions against IUGR‐induced hepatic injury.

## 4 Data and Code Availability

The datasets generated in this study are available in the following databases: scRNA‐seq data: https://www.ncbi.nlm.nih.gov/geo/query/acc.cgi?acc=GSE222949, RNA‐seq data: https://www.ncbi.nlm.nih.gov/geo/query/acc.cgi?&acc=GSE233802, and lipidomic data: www.ebi.ac.uk/metabolights/MTBLS6842. We have constructed an interactive shiny‐app web tool for better exploration (http://biomamba.com:34038/IUGR.wys.shiny/). Referenced RNA‐seq and scRNA‐seq data: https://www.ncbi.nlm.nih.gov/bioproject/PRJNA597972; https://www.ncbi.nlm.nih.gov/geo/query/acc.cgi?acc=GSE106512; https://www.ncbi.nlm.nih.gov/geo/query/acc.cgi?acc=GSE136103; https://www.ebi.ac.uk/biostudies/arrayexpress/studies/E‐MTAB‐7407. The article and its Supporting Information files contain all other relevant data supporting the main findings of the study. All codes for processing the scRNA‐seq data have been deposited in GitHub and are available at the following URL: https://github.com/yusenWei/IUGR/.

## 5 Experimental Section

### Collection of Neonatal Venous Blood

Venous blood was collected through femoral or elbow vein puncture, as deemed appropriate. The neonate was placed in a supine position with the thighs abducted, and the femoral artery was identified by palpation. The needle was then inserted vertically at a distance of 0.5 cm after sterilization. Serum was obtained by centrifugation at 3500×g for 5 min at 4 °C for determining biochemical indicators. All procedures were performed in accordance with the ethical review and approval document (no. 2023‐KLS‐232‐02).

### Animals

Sixteen pairs of piglets, comprising IUGR and NBW piglets, were selected based on birth weight, ensuring that one from each litter and of the same sex were chosen (male n = 8; female n = 8). Thirty‐two male piglets, representing the male IUGR and NBW piglet cohort, were selected and evenly distributed among four sows for lactation (n = 8).

### ScRNA‐seq

Liver tissues were removed from MACS Tissue Storage Solution and processed to generate a single‐cell suspension. The single‐cell suspension concentration was adjusted to 1000 cells µl^−1^ for the preparation of the scRNA‐Seq libraries using a 10X Genomics Chromium Controller Instrument and Chromium Single Cell 3′ V3.1 Reagent Kits (10X Genomics, Pleasanton, CA). All libraries were sequenced using an Illumina NovaSeq 6000 sequencer (Illumina, San Diego, CA) on a 150 bp paired‐end run.

### Flow Cytometry

Cells were blocked with FC blocking (made in the laboratory) for 20 min on ice and followed by surface staining using a panel of antibodies on ice in the dark for 30 min.

### ScRNA‐seq Data Processing

The fastp software was used to filter adaptor sequences and remove low‐quality reads. Any ambient RNA background was evaluated for each library and removed. Downstream analysis, including data processing, clustering, dimensional reduction, differential expression determination, subclustering analysis, functional enrichment, cellular regulatory‐network, cellular trajectory, and cellular interaction, was performed.

### Lipid Metabolomics

Each sample of 20 mg were homogenized with 1 ml of a mixture (MTBE/methanol (3:1, *v*/*v*) and internal standard mixture with a mixer mill with zirconia beads for lipid extraction. The supernatant (300 µl) was obtained by centrifugation at 4 °C at 12000×g for 10 min. The supernatant was dried with nitrogen and reconstituted in acetonitrile/isopropanol (1:9, v/v) for UPLC‐MS/MS analysis. Ultra Performance Liquid Chromatography (UPLC) system (ExionLCTM AD, SCIEX, Framingham, MA) with Tandem Mass Spectrometry (QTRAP 6500+) was applied. Chromatographic columns (AccucoreTM C30, 2.6 µm, 2.1 mm × 100 mm i.d.) (Thermo Fisher Scientific, Waltham, MA) for separation of the lipids were used.

### Lipidomic Data Analysis

SCIEX MultiQuant software (v3.0.3) was performed to integrate and correct characteristic fragment peaks of each lipid, resulting in a lipid data matrix. The lipid data was log transformed and zero‐centered scaled to prepare for downstream analysis.

### Construction of APOA4‐KO HepG2 Cell Line

The APOA4 gene was successfully ablated using CRISPR‐Cas9 mediated by CRISPR‐Cas9 RNP (Haixing Bioscience), which contained expression cassettes for hSpCas9 and chimeric guide RNA.

### Data Analysis

Significant differences were assessed using a two‐sided paired or unpaired Student's t‐test and unpaired Wilcoxon rank‐sum test where indicated. The data from three independent experiments were presented as means±SD. The presence of a “*” sign indicates that the *P* value did not exceed 0.05 which was considered statistically significant.

### Abbreviations

AST/ALT, aminotransferase/alanine aminotransferase; ALB, albumin; ALP, alkaline phosphatase; CHE, cholinesterase; CAR, carnitine; DEGs, differentially expressed genes; Endo, endothelial cells; E2, estradiol; GLOB, globulin; GO, Gene Ontology; Hep, hepatocytes; IGF‐1, insulin‐like growth factor I; IUGR, intrauterine growth restriction; KEGG, Kyoto Encyclopedia of Genes and Genomes; LBW, low birth weights; NNMF, non‐negative matrix factorization; TG, triglyceride; TP, total protein; scRNA‐seq, single‐cell RNA sequencing; UMAP, uniform manifold approximation and projection; VLDL, very low‐density lipoprotein.

## Conflict of Interest

The authors declare no conflict of interest.

## Supporting information

Supporting Information

Supplemental Table 1

Supplemental Table 2

Supplemental Table 3

Supplemental Table 4

Supplemental Table 5

Supplemental Table 6

Supplemental Table 7

Supplemental Table 8

Supplemental Table 9

Supplemental Table 10

Supplemental Table 11

Supplemental Table 12

Supplemental Table 13

Supplemental Table 14

Supplemental Table 15

Supplemental Table 16

Supplemental Table 17

## Data Availability

The data that support the findings of this study are openly available in GEO∖metabolights at https://www.ncbi.nlm.nih.gov/geo/query/acc.cgi?acc = GSE222949∖https://www.ncbi.nlm.nih.gov/geo/query/acc.cgi?&acc = GSE233802∖www.ebi.ac.uk/metabolights/MTBLS6842, reference number 222949.
