# Supplementary material for: Sexually Dimorphic Response to Hepatic Injury in Newborn Suffering from Intrauterine Growth Restriction
Source: Adv Sci (Weinh). 2024 Jun 13;11(30):2403095. doi: 10.1002/advs.202403095 (PMC11321654; doi:10.1002/advs.202403095)
Supplement: Supplementary file 1 — Supporting Information [file ADVS-11-2403095-s001.docx]

Supporting Information

Sexually Dimorphic Response to Hepatic Injury in Newborn Suffering from Intrauterine Growth Restriction

*Yu-Sen Wei, Wen-Jie Tang, Pei-Yu Mao, Jiang-Di Mao, Zhi-Xiang Ni, Kang-Wei Hou, Teresa G. Valencak, Da-Ren Liu, Jun-Fang Ji, and Hai-Feng Wang**

**Supporting Information Text**

**Methods**

**Animals.** Our study involved approximately 60 healthy sows (Large White breed) in their second parity that delivered at the same time. Sixteen pairs of piglets (female & male, indicated as ♀ and ♂, respectively, Large White breed), consisting of IUGR (birth weight < 1.0 kg) and NBW (birth weight > 1.5 kg & < 1.8 kg) piglets, were selected mainly based on birth weight with head shape as a reference. We selected piglets from each litter and belonging to the same sex (male n=8; female n=8). Thirty-two male piglets, representing the male IUGR and NBW piglet cohort, were selected and evenly distributed among four sows for lactation (n=8). Within this cohort, two litters received icariin from 7d to 28d through oral administration. The piglets received standard feed without any additional interventions. One week after birth and after 28d, piglets were euthanized with an intraperitoneal injection of pentobarbital sodium (25 mg/kg body weight) after measuring their body weights and collecting blood samples from the *Vena cava anterior*. Serum was obtained by centrifugation at 3000×g for 15min at 4℃, and stored at -80℃ until later analyses. The left lateral lobe of the liver was carefully prepared and preserved in MACS Tissue Storage Solution (Miltenyi Biotec, 130-100-008) until later processing. Liver segments were fixed in 4% paraformaldehyde (PFA), an optimal cutting temperature compound (OCT compound), or 2.5% glutaraldehyde for histology. Additionally, liver samples were frozen in liquid nitrogen and stored at -80℃ for extracting lipids, RNA, and protein.

**Serum assays.** Serum concentration of ALP, ALT, AST, CHE, TP, ALB, GLOB, HDL-C, LDL-C, TG, TCHOL, TBA, TBIL, GLU and LDH were measured using a BX-4000 automatic biochemical analyzer (Sysmex, Kobe, Japan). The quantification of liver TG and cell lines was conducted using a triglyceride content determination kit (Solarbio Science & Technology, China, BC0620).

**RNA-seq data processing.** Total RNA from samples was isolated and purified using the TRIzol reagent (Invitrogen, Carlsbad, USA). After purification of poly (A) RNA, reverse transcription, and amplification, cDNA libraries were generated using NEBNext® Ultra^TM^ RNA Library Prep Kit for Illumina (NEB, #E7770, USA) and sequenced with 2×150bp paired-end sequencing (PE150) on an Illumina Novaseq™ 6000 (Illumina, San Diego, CA).

Raw data from fastq files were processed using fastp software (v0.23.1) with default parameters to obtain clean reads by removing adaptor contamination, low-quality bases, and undetermined bases. The clean reads were then mapped to the Ensembl *Sus scrofa* 11.1 reference genome (release 105) or the Ensembl *GRCh* 38.p13 (release 109) using HISAT2^[1]^ software (v2.1.0). The “featureCounts” function in the subread package (v2.0.2) was used to quantify mRNA expression levels. Differentially expressed mRNAs were selected based on log_2_ FC (|log_2_ FC| ≥ 1.0) and models derived from a negative binomial distribution (*P* value < 0.05) using the DESeq2^[2]^ package (v1.38.3), and used for functional enrichment analysis. The co-expression network and concomitant temporal clustering of genes were identified using the WGCNA^[3]^ package (v1.72-1) and Mfuzz^[4]^ package (v2.60.0).

**ScRNA-seq.** Liver tissues were removed from MACS Tissue Storage Solution and processed to generate a single-cell suspension. Briefly, samples were washed with Roswell Park Memorial Institute (RPMI) 1640 Medium, sliced into small pieces (approximately 1mm^3^) on ice, and enzymatically digested with 2.0 mg/ml collagenase Ⅳ (Sigma, 9001-12-1), 0.25 mg/ml HAase (Sigma. 3732633-3) and 20 μl/ml DNase Ⅰ (Sigma, 9003-98-9) at 37℃ with shaking for 1 h. Following digestion, samples were sieved through a 70μm stacked cell strainer and centrifuged at 400×g for 10 min. After removing the supernatant, cell pellets were suspended in ACK Lysis Buffer (Homemade) to lyse red blood cells. After washing with PBS containing 0.05% BSA, cell pellets were resuspended in PBS containing 0.05% BSA and layered on 36% percoll (GE, 17089101) by centrifuging at 400×g for 30 min to lyse cellular debris. Dissociated single cells were stained with AO/PI for viability assessment using a Countstar Rigel S2 Fluorescence Cell Analyzer (Ruiyu Biotech Co., Ltd., Shanghai, China).

The single-cell suspension concentration was adjusted to 1000 cells/μl for the preparation of the scRNA-Seq libraries using a 10X Genomics Chromium Controller Instrument and Chromium Single Cell 3’ V3.1 Reagent Kits (10X Genomics, Pleasanton, CA). Approximately 8,000 cells were loaded into each channel to generate single-cell Gel Bead-In-Emulsions (GEMs), resulting in an expected mRNA barcoding of 6,000 single cells for each sample. The following cDNA amplification and library construction steps followed the manufacturer’s instructions. All libraries were sequenced using an Illumina NovaSeq 6000 sequencer (Illumina, San Diego, CA) on a 150 bp paired-end run.

**Flow cytometry.** Approximately one million cells per sample were plated for flow cytometry analysis. Cells were blocked with FC blocking (madein the laboratory) for 20 min on ice and followed by surface staining using a panel of antibodies on ice in the dark for 30 min. After washing with and resuspending in FACS buffer (made in the laboratory), cells were analysed on a Mindray Flow Cytometer (BriCyte E6). Data analysis was performed using FlowJo v10.0.7. Antibodies are provided in Table S17, Supporting Information.

**ScRNA-seq data processing.** We applied the fastp software with default parameters to filter adaptor sequences and remove low-quality reads, generating “clean” data. Gene expression matrices were obtained by aligning reads to the Ensembl *Sus scrofa* 11.1 reference genome (release 105) using the CellRanger software (v6.1.1). Any ambient RNA background was evaluated for each library and removed using the SoupX^[5]^ package (v1.6.2). The pipeline of Seurat^[6]^ package (v4.1.6) was used for downstream analysis, including data processing, clustering, dimensional reduction and differential expression determination. Cell transcriptomes with fewer than 200 or more than 6,000 genes or the mitochondria UMI (unique molecular identifier) rate above 20% were filtered out ahead of analysis. The UMI counts were transformed and normalized, and cell cycle effects were also adjusted by regressing out the G2M and S phase gene expression scores using the “SCTransform()” function. The “IntegrateData()” function and Harmony^[7]^ package (v1.0) were applied for de-batch integration of data. A PCA was constructed based on the SCT matrix with the top 2000 high variable genes using the “RunPCA()” function and the top 30 principals were used for UMAP^[8]^ construction using “RunUMAP” function. The biomaRt^[9]^ package (v2.54.0) was used to transfer homologous genes between human and pig.

**Subclustering analysis.** Unsupervised cell clustering was done using a graph-based cluster method. Marker genes were calculated by the “FindAllMarkers()” function with the MAST^[10]^ package (v1.24.1) based on criteria such as a cell expression percentage > 25%, log_2_FoldChange > 0.25, and adjustment q value < 0.01. After cluster annotation, DEGs in each cell type between NBW and IUGR samples were identified by the “FindMarker” function with the MAST algorithm. To explore heterogeneity within cell type in detailed, clusters from the same source were selected using the “subset()” function for re-UMAP analysis, graph-based clustering, and marker analysis. In addition, the NMF^[11]^ package (v0.25) was used to identify variable expression programs among subtypes based on the number of factors set. The top 50 genes for each cluster were defined as the meta-signature for cell type identification.

**Functional enrichment analysis.** DEGs between NBW and IUGR samples were obtained from Metascape^[12]^ (http://metascape.org) for GO biological process analysis. KEGG analysis was carried out using the clusterProfiler^[13]^ package (v4.0). The KEGG terms of selected genes were enriched in the database “org.Ss.eg.db” using the “enrichKEGG()” function. The GSVA^[14]^ package (v1.46.0) was used to assess the relative pathway activities in the inflammatory subtype based on the hallmark gene sets in the MSigDB database (https://www.gSea-msigdb.org/gsea/msigdb). Self-made specific gene sets (Table S16, Supporting Information) were scored using the “AddModuleScore()” function in the Seurat package.

**Cellular regulatory-network analysis.** The *pySCENIC*^[15]^ Python package (v0.12.1) was used to infer TF-target interactions. There are three main commands, including *grn*, which infers the gene-gene co-expression relationship between TFs and their potential targets using the “grnboost2” algorithm, *ctx* that identifies the regulons that contain one TF and its target genes enriched for the motifs of the TF, and *aucell*, which calculates the activities of the regulons for each cluster. The TF gene list of *Sus scrofa* was downloaded from the animalTFDB3.0^[16]^ (http://bioinfo.life.hust.edu.cn/AnimalTFDB/). The specificity scores of the regulons (RSS) in each cluster were calculated and ordered using the “regulon_specificity_scores()” function and visualized using the Seurat and ggplot2 packages (v3.3.0).

**Cellular trajectory analysis.** The single-cell pseudotime trajectories for the CD4+ T cell and myeloid cells were generated using the Monocle2^[17]^ package (v2.26.0). Genes with high dispersion selected by Monocle2 were applied for dimension reduction using the DDRTree method. The cells were ordered and visualized with the “plot_cell_trajectory()” function. Genes changing along pseudotime were calculated by the “differentialGeneTest()” function and visualized with the “plot_genes_in_pseudotime” function and “plot_pseudotime_heatmap” function. Genes were clustered into subgroups according to their expression patterns. Branch expression analysis (BEAM) was used to identify genes separating cells into branches, and the “plot_genes_branched_heatmap()” function was applied for visualization. To confirm the trajectory result, the Monocle3^[18]^ package (v1.2.7) was applied to the mini-cluster expression matrix and UMAP embedding, preserving local relations of cells. The functions “cluster_cells” and “learn_graph” were used to divide the mini-cluster into large and well-separated groups called partitions and fit a principal graph within each partition. The principal graph was displayed as a “skeleton line” on UMAP, indicating the differentiation trajectory.

We also used the *ScVelo*^[19]^ Python package (v0.2.5) to calculate RNA velocity in subtypes of Kupffer cells. RNA velocity estimation was implemented using the “scv.tl.velocity” function with the mode set to “dynamical”. The “scv.tl.velocity_graph()” function was used to construct a velocity graph representing transition probabilities among cells and the “scv.pl.velocity_embedding_stream()” function displayed the velocity graph embedding the RNA velocities into the previously generated UMAP.

**Cellular interaction analysis.** The *CellPhoneDB*^[20]^ python package (v3.1.0) with default parameters was used to explore intercellular communication in the hepatic microenvironment. The number of L-R pairs among each cell type was visualized using the “heatmap_plot” function and differential L-R pairs were visualized using the “dot_plot” function. The CellChat^[21]^ package (v1.1.3) with default parameters was used to investigate the altered communication between the inflammatory subtype and non-immune cells in IUGR samples. Differential strength network and information flow were visualized using the “netVisual_diffInteraction” and “rankNet” functions, and the cell-cell communication network was visualized using the “netVisual_aggregate” function. A centrality score was computed and visualized using the “netAnalysis_signalingRole_network” function, and the relative contribution of each L-R pair was visualized using the “netAnalysis_contribution” function.

**Histopathology analysis.** Paraffin sections of liver tissue were dewaxed and rehydrated in staining solution (hematoxylin and eosin, periodic acid and Schiff’s solution, 2% potassium ferrocyanide, and 2% hydrochloric acid). Cryosections were dehydrated by isopropanol and fixed in 4% PFA, and were incubated in Oil Red O staining solution. The sections were washed and dehydrated and then mounted using Eukitt.

**Transmission electron microscopy.** After being fixed with 2.5% glutaraldehyde for 6 h, liver specimens were postfixed with 1% OsO4 for 2h and rinsed three times with the PBS buffer (0.1M, pH=7.0) for 15 mins at each step. Then, specimens were subjected to a graded series of dehydration processes, including ethanol (30%, 50%, 70%, 80%) and acetone (90%, 95%, 100%). Before being embedded and heated for 8h at 65℃, specimens were placed in a mixture of acetone and Spurr resin (1:1 *v*/*v* for 1h, 1:3 *v*/*v* for 3 h) and pure Spurr resin overnight. A Leica EM UC7 (Leica, AUT) was used to cut the samples. Finally, they were stained by uranyl acetate and alkaline lead citrate for 5 to 10 min, respectively and observed using a H-7650 TEM (Hitachi, JPN).

**Immunohistochemistry and immunofluorescence staining.** After being dewaxed and dehydrated, samples were processed with 3% hydrogen peroxide to terminate endogenous peroxidase activity. For IF staining, they were subjected to antigen retrieval by EDTA compound (1 mol/L, pH=6.0) and permeabilized with normal goat serum (CWBIO, CW0130) for 20 min at 25℃. After incubation with the primary detection antibody and secondary antibody, nuclei were counterstained using DAPI (CST, #8961) for 5 min. For IHC staining, the samples were subjected to antigen retrieval by AR9 buffer (pH=6.0) at 96℃ and permeabilized with normal goat serum for 20 min at 25℃. After incubation with the primary detection antibody and secondary antibody, the samples were visualized using diaminobenzidine (DAB). Then nuclei were counterstained using hematoxylin for 2 min. All antibodies were listed in Table S17, Supporting Information.

**Imaging.** Images of fluorescence and bright-field were performed on PANNORAMIC MIDI II (3DHISTECH Ltd., HUN)**.** TEM images were obtained using a Gatan 830 CCD camera (Gatan, USA). Image analysis was performed using CaseViewer (v2.4, 3DHISTECH Ltd., HUN) and ImageJ software (National Institute of Health, USA).

**Lipid metabolomics.** 20 mg from each sample were homogenized with 1 ml of a mixture (MTBE/methanol (3:1, *v*/*v*) and internal standard mixture with a mixer mill with zirconia beads for lipid extraction. After vortexing for 2 min and emulsification by sonication for 5 min, 200 μl of water was added and the samples were vortexed for 1 min. The supernatant (300 μl) was obtained by centrifugation at 4℃ at 12,000×g for 10 min. The supernatant was dried with nitrogen and reconstituted in acetonitrile/isopropanol (1:9, v/v) for UPLC-MS/MS analysis.

Ultra Performance Liquid Chromatography (UPLC) system (ExionLCTM AD, SCIEX, Framingham, MA) with Tandem Mass Spectrometry (QTRAP® 6500+) was applied. Chromatographic columns (Accucore^TM^ C30, 2.6μm, 2.1mm×100mm i.d.) (Thermo Fisher Scientific, Waltham, MA) for separation of the lipids were used. The solvent system contained a mobile phase A (acetonitrile/water, 60/40, *v*/*v*, 0.1% formic acid, 10 nmol/L ammonium formate) and mobile phase B (acetonitrile/isopropanol, 10/90, *v*/*v*, 0.1% formic acid, 10 nmol/L ammonium formate). Gradient elution at a flow rate of 0.35 ml/min was performed at 45℃ as follows: A/B (80:20, *v*/*v*) at 0 min, A/B (70:30, *v*/*v*) at 2 min, A/B (40:60, *v*/*v*) at 4 min, A/B (15:85, *v*/*v*) at 9 min, A/B (10:90, *v*/*v*) at 14 min, A/B (5:95, *v*/*v*) at 15.5 min, A/B (5:95, *v*/*v*) at 17.3 min, A/B (80/20, *v*/*v*) at 17.5 min, A/B (80/20, *v*/*v*) at 20 min.

Subsequently, the effluent was linked with an ESI-triple quadrupole-linear ion trap (QTRAP)-MS for detection and quantification in the multiple reaction monitoring (MRM) mode using SCIEX Analyst software (v1.6.3). Parameters were as follows: ion source temperature, 500℃; source gas1, 45 psi; gas2, 55 psi; curtain gas, 35 psi.

**Lipidomic data analysis.** Based on the internal standard method, lipid identification was achieved from the acquired lipids, which were compared and matched using the MetWare database (http://www.metware.cn/) (MetWare Biological Science and Technology Co., Ltd., Wuhan, China), including retention time, accurate precursor ions/product ion information, and MS/MS spectrum patterns. The SCIEX MultiQuant software (v3.0.3) was used to integrate and correct characteristic fragment peaks of each lipid, resulting in a lipid data matrix. The lipid data was log transformed and zero-centered scaled to prepare for OPLS-DA analysis. The “OPLSR.Anal()” function in the MetaboAnalyst^[22]^ package (v2.0.0) was applied to construct the OPLS-DA model. The model calculated the value of variable importance in projection (VIP) values. Differential lipid metabolites between male NBW and IUGR individuals were determined based on a combination of (VIP ≥ 1) and absolute log_2_ FC (|log_2_ FC| ≥ 1.0). Pearson coefficient was computed for correlation analysis. ROC curve analysis was done using the pROC^[23]^ package (v1.16.2) to precisely discriminate differential metabolites.

**RT-qPCR.** Total RNA was isolated using the *SteadyPure* Universal RNA Extraction Kit Ⅱ (AGBIO, AG21022) according to the manufacturer’s protocol. The RNA concentration and quality were determined with a Nanodrop 2000 spectrophotometer (ThermoFisher Scientific, Switzerland) and the same mass was transcribed into cDNA using the *Evo M-MLV* Mix Kit with gDNA Clean for qPCR (AGBIO, AG11728). The cDNA was diluted to 2 ng/µl, and qPCR was performed with the SYBR Green Premix Pro Taq HS Qpcr Kit (AGBIO, AG11701) on a CFX96 Real-Time PCR thermocycler (Bio-Rad, California, UK). The relative expression levels for RT-qPCR were calculated using the 2^-(△△CT). The primer sequences are listed in Table S17, Supporting Information.

**Enzyme-linked immunosorbent assay.** Levels of IGF-1, E2, IL-6, TNF-α, VLDL and APOA4 were determined using an ELISA Kit according to the manufacturer. All ELISA Kits are listed in Table S17, Supporting Information.

**Construction of APOA4-KO HepG2 cell line.** The APOA4 gene was successfully ablated using CRISPR-Cas9 mediated by CRISPR-Cas9 RNP (Haixing Bioscience), which contained expression cassettes for hSpCas9 and chimeric guide RNA. Two guiding RNAs were selected from the website (http://crispr.mit.edu) to target exon 1~exon 3 of the APOA4 gene. The plasmid carrying the guide RNA sequence was electro-transfected into cells using the Neon transfection system (ThermoFisher Scientific, Waltham, MA) following the manufacturer's instructions. After two days, single colonies were transferred into 96-well plates. To confirm the presence of insertions or deletions (indels) in the APOA4 targeted clones, genomic DNA was isolated using the Quick-DNA Miniprep kit (Zymo Research, D3025), and PCR amplification was performed using 2×Taq Master Mix (Dye Plus) (Vazyme, P112) with primers flanking the exon. The amplified products were separated by electrophoresis on a 1.5% agarose gel. Plasmids were isolated from 8-10 single colonies and subjected to Sanger sequencing (GENEWIZ, China) for verification. Clones with mutations in both alleles were selected for further analysis. The cell lines generated using the aforementioned strategy included gene (+/+), gene (+/-), and gene (-/-) cells. All the clones were maintained under the same conditions as the parental cells.

**Cell culture and cell induction.** The normal and APOA4-KO HepG2 cell lines were cultured in complete medium for HepG2 (Haixing Bioscience, TCH-G196). The THP-1 cell line was cultured in complete medium for THP-1 (Haixing Bioscience, TCH-G361). For induction of hypoxia, a cell culture was used at 1% oxygen for 24 or 48h in a Tri-gas Incubator (Thermo Fisher Scientific, Waltham, MA). The culture media of the HepG2 cell line were collected under both hypoxic and normoxic culture conditions. The THP-1 cell line was differentiated into macrophages using 100 ng/ml of PMA for 48h. M1 polarization was induced by treating the cells with a combination of 100 ng/ml of LPS and 20 ng/ml of IFN-γ for 48h.

**Cell cycle assay and colony formation assay.** Cell cycle distribution was detected using a NovoCyte Flow Cytometer (Agilent, USA). Cells were fixed in 70% cold ethanol for 12h and stained with 500 μL propidium iodide. Analysis of the results was conducted using NovoExpress software Version xx (Agilent, USA). For the colony formation assay, cells were seeded in 6-well plates in triplicate at densities of 50/100/200 cells per well and cultured for two weeks to allow for colony formation. Colonies were fixed with PFA and stained with 0.1% crystal violet. Colony counting was performed using ImageJ software.

**Western blotting.** Total protein lysates were obtained using RIPA buffer (Beyotime, P0013B) supplemented with protease and phosphatase inhibitors (Beyotime, P1010; P1050). Protein concentration was determined with the BCA Protein Assay Kit (Beyotime, P0010S). Equal masses of proteins were separated by 10% polyacrylamide gel electrophoresis and blotted onto polyvinylidene fluoride membranes. Upon blocking with QuickBlock™ Blocking Buffer for Western Blotting (Beyotime, P0252), the membranes were incubated with primary antibodies at 4 ℃ o/n. According to the location of the marker, the gel was cut into sections including target protein with different molecular weights and internal reference β-actin protein. For two proteins with similar molecular weights, the membrane after blotting with one antibody was washed with stripping buffer (NCM biotech, WB6500) before incubation with another primary antibody. Incubation with suitable secondary antibodies was performed at RT for 1h. The signal was detected using the BeyoECL Star (Beyotime, P0018AS). Protein bands of interest were visualized using a gel-documentation system (Thermo Fisher Scientific, Waltham, MA) and densitometric analysis was performed using ImageJ software. Antibodies are listed in Table S17, Supporting Information.

**Data analysis.** High-throughput data were analysed statistically using the respective R packages. Significant differences were assessed using a two-sided paired or unpaired Student’s *t*-test and unpaired Wilcoxon rank-sum test where indicated. The data from at least three independent experiments were presented as means±SD. The presence of a “*” sign indicates that the *P* value did not exceed 0.5, which was considered statistically significant. All statistical analyses and graph generation were conducted in R (v4.1.2) and GraphPad Prism (v8.0).

References

[1] D. Kim, J. M. Paggi, C. Park, C. Bennett, S. L. Salzberg, *Nature Biotechnology* **2019**, *37* (8), 907, https://doi.org/10.1038/s41587-019-0201-4.

[2] M. I. Love, W. Huber, S. Anders, *Genome Biol* **2014**, *15* (12), 550, https://doi.org/10.1186/s13059-014-0550-8.

[3] P. Langfelder, S. Horvath, *BMC Bioinformatics* **2008**, *9* (1), 559, https://doi.org/10.1186/1471-2105-9-559.

[4] L. Kumar, E. F. M, *Bioinformation* **2007**, *2* (1), 5, https://doi.org/10.6026/97320630002005.

[5] M. D. Young, S. Behjati, *Gigascience* **2020**, *9* (12), https://doi.org/10.1093/gigascience/giaa151.

[6] Y. Hao, S. Hao, E. Andersen-Nissen, W. M. Mauck, 3rd, S. Zheng, A. Butler, M. J. Lee, A. J. Wilk, C. Darby, M. Zager, P. Hoffman, M. Stoeckius, E. Papalexi, E. P. Mimitou, J. Jain, A. Srivastava, T. Stuart, L. M. Fleming, B. Yeung, A. J. Rogers, J. M. McElrath, C. A. Blish, R. Gottardo, P. Smibert, R. Satija, *Cell* **2021**, *184* (13), 3573, https://doi.org/10.1016/j.cell.2021.04.048.

[7] I. Korsunsky, N. Millard, J. Fan, K. Slowikowski, F. Zhang, K. Wei, Y. Baglaenko, M. Brenner, P.-r. Loh, S. Raychaudhuri, *Nature Methods* **2019**, *16* (12), 1289, https://doi.org/10.1038/s41592-019-0619-0.

[8] E. Becht, L. McInnes, J. Healy, C.-A. Dutertre, I. W. H. Kwok, L. G. Ng, F. Ginhoux, E. W. Newell, *Nature Biotechnology* **2019**, *37* (1), 38, https://doi.org/10.1038/nbt.4314.

[9] S. Durinck, Y. Moreau, A. Kasprzyk, S. Davis, B. De Moor, A. Brazma, W. Huber, *Bioinformatics* **2005**, *21* (16), 3439, https://doi.org/10.1093/bioinformatics/bti525.

[10] G. Finak, A. McDavid, M. Yajima, J. Deng, V. Gersuk, A. K. Shalek, C. K. Slichter, H. W. Miller, M. J. McElrath, M. Prlic, P. S. Linsley, R. Gottardo, *Genome Biology* **2015**, *16* (1), 278, https://doi.org/10.1186/s13059-015-0844-5.

[11] R. Gaujoux, C. Seoighe, *BMC Bioinformatics* **2010**, *11* (1), 367, https://doi.org/10.1186/1471-2105-11-367.

[12] Y. Zhou, B. Zhou, L. Pache, M. Chang, A. H. Khodabakhshi, O. Tanaseichuk, C. Benner, S. K. Chanda, *Nat Commun* **2019**, *10* (1), 1523, https://doi.org/10.1038/s41467-019-09234-6.

[13] S. Hänzelmann, R. Castelo, J. Guinney, *BMC Bioinformatics* **2013**, *14*, 7, https://doi.org/10.1186/1471-2105-14-7.

[14] T. Wu, E. Hu, S. Xu, M. Chen, P. Guo, Z. Dai, T. Feng, L. Zhou, W. Tang, L. Zhan, X. Fu, S. Liu, X. Bo, G. Yu, *Innovation (Camb)* **2021**, *2* (3), 100141, https://doi.org/10.1016/j.xinn.2021.100141.

[15] S. Aibar, C. B. González-Blas, T. Moerman, V. A. Huynh-Thu, H. Imrichova, G. Hulselmans, F. Rambow, J.-C. Marine, P. Geurts, J. Aerts, J. van den Oord, Z. K. Atak, J. Wouters, S. Aerts, *Nature Methods* **2017**, *14* (11), 1083, https://doi.org/10.1038/nmeth.4463.

[16] H. Hu, Y. R. Miao, L. H. Jia, Q. Y. Yu, Q. Zhang, A. Y. Guo, *Nucleic Acids Res* **2019**, *47* (D1), D33, https://doi.org/10.1093/nar/gky822.

[17] X. Qiu, A. Hill, J. Packer, D. Lin, Y. A. Ma, C. Trapnell, *Nat Methods* **2017**, *14* (3), 309, https://doi.org/10.1038/nmeth.4150.

[18] J. Cao, M. Spielmann, X. Qiu, X. Huang, D. M. Ibrahim, A. J. Hill, F. Zhang, S. Mundlos, L. Christiansen, F. J. Steemers, C. Trapnell, J. Shendure, *Nature* **2019**, *566* (7745), 496, https://doi.org/10.1038/s41586-019-0969-x.

[19] V. Bergen, M. Lange, S. Peidli, F. A. Wolf, F. J. Theis, *Nature Biotechnology* **2020**, *38* (12), 1408, https://doi.org/10.1038/s41587-020-0591-3.

[20] M. Efremova, M. Vento-Tormo, S. A. Teichmann, R. Vento-Tormo, *Nature Protocols* **2020**, *15* (4), 1484, https://doi.org/10.1038/s41596-020-0292-x.

[21] S. Jin, C. F. Guerrero-Juarez, L. Zhang, I. Chang, R. Ramos, C.-H. Kuan, P. Myung, M. V. Plikus, Q. Nie, *Nature Communications* **2021**, *12* (1), 1088, https://doi.org/10.1038/s41467-021-21246-9.

[22] Z. Pang, J. Chong, G. Zhou, D. A. de Lima Morais, L. Chang, M. Barrette, C. Gauthier, P.-É. Jacques, S. Li, J. Xia, *Nucleic Acids Research* **2021**, *49* (W1), W388, https://doi.org/10.1093/nar/gkab382.

[23] X. Robin, N. Turck, A. Hainard, N. Tiberti, F. Lisacek, J.-C. Sanchez, M. Müller, *BMC Bioinformatics* **2011**, *12* (1), 77, https://doi.org/10.1186/1471-2105-12-77.

**Supplementary Figures & Tables**

**Figure S1. Phenotypic differences between IUGR and NBW male piglets one week after birth.** a-c) Serum parameters including LDL-C and TCHOL (a), TBA and TBIL (b), GLU and LDH (c). d) Relative organ weight indices including kidney, lung, heart, and spleen. Two-sided paired Student’s t-test (n=8, a-d) (**P* < 0.05, ns *P* > 0.05). LDL-C, low-density lipoprotein cholesterol; TCHOL, total cholesterol; TBA, total biliary acid; TBIL, total bilirubin; GLU, glucose; LDH, lactic dehydrogenase.

**
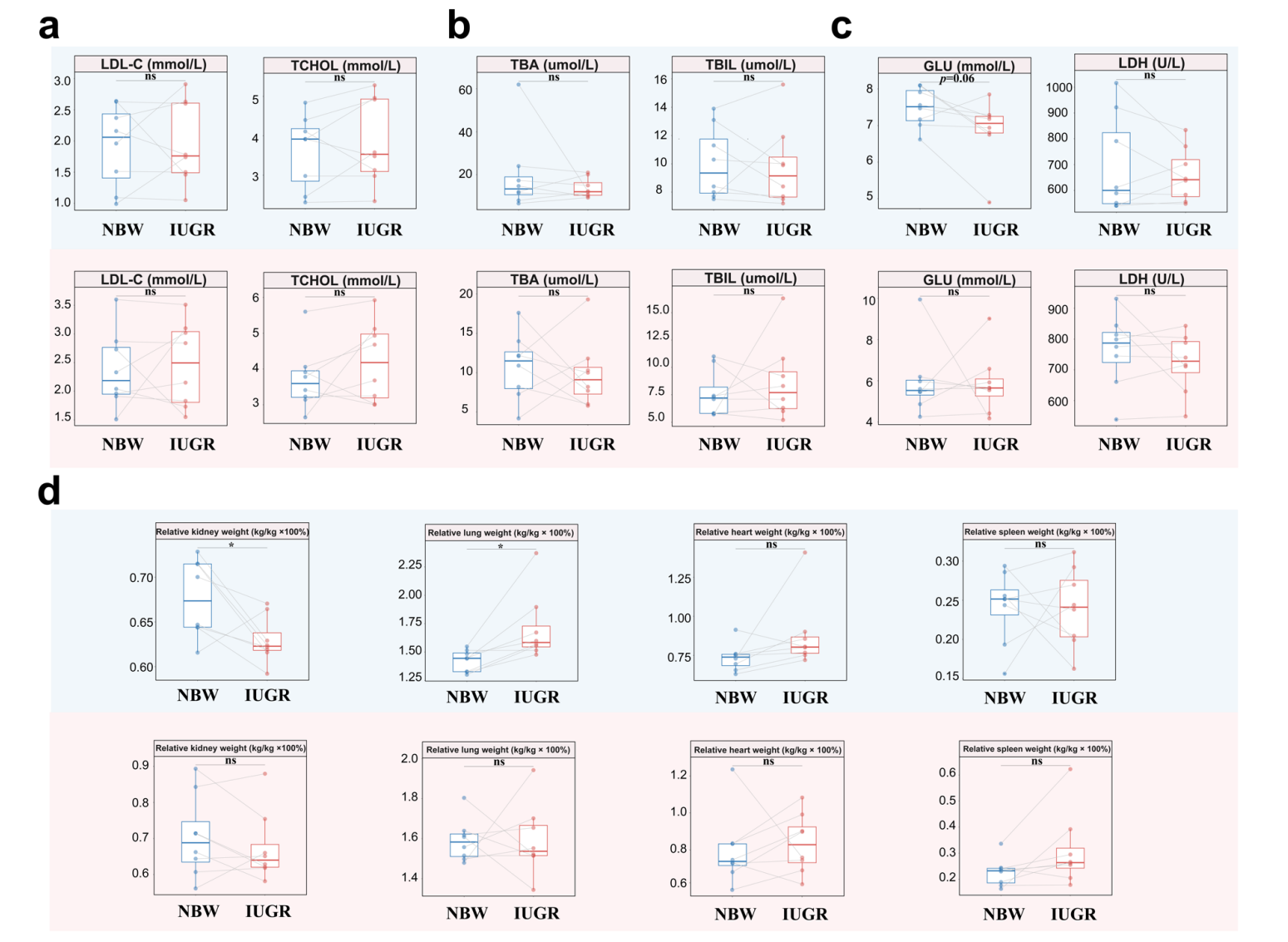
**

**Figure S2. Overview of RNA-seq from IUGR and NBW from public data.** a) Transcriptional profiling of the DGEs between IUGR and NBW piglets from both genders one week after birth. b) Comparison of mRNA expression of EPO and GAPDH in livers of NBW and IUGR piglets from both genders (n=8). c) Classification of the KEGG terms enriched between IUGR and NBW piglets females one week after birth. d) Detailed function profiling of the KEGG terms enriched between IUGR and NBW pigs in males on Day 7 and Day 150. e) Integrated analysis about genome-wide distribution of DEGs between IUGR and NBW pigs in males on Day 7 and Day 150. f) Recorded litter sizes on the two farms and comparison of the percentage of weak litters in different genders. Two-sided paired Student’s t-test (b) (**P* < 0.05).
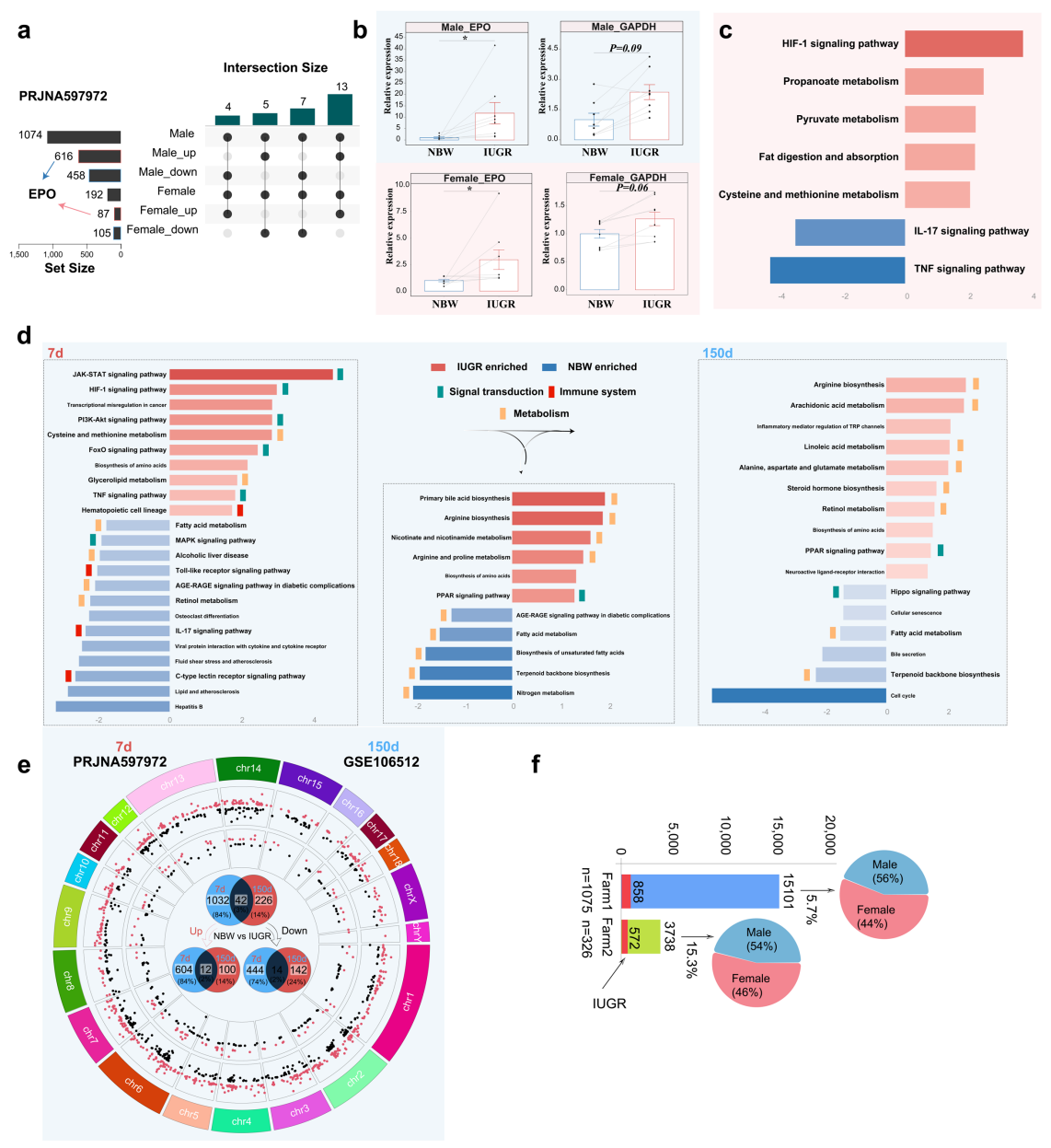


**Figure S3. Overview of experimental design** created with BioRender.com**.** Single-cell sequencing for gathering cellular-level data perturbed by IUGR, and lipidomics for pinpointing classes of altered lipids in male livers affected by IUGR.

**
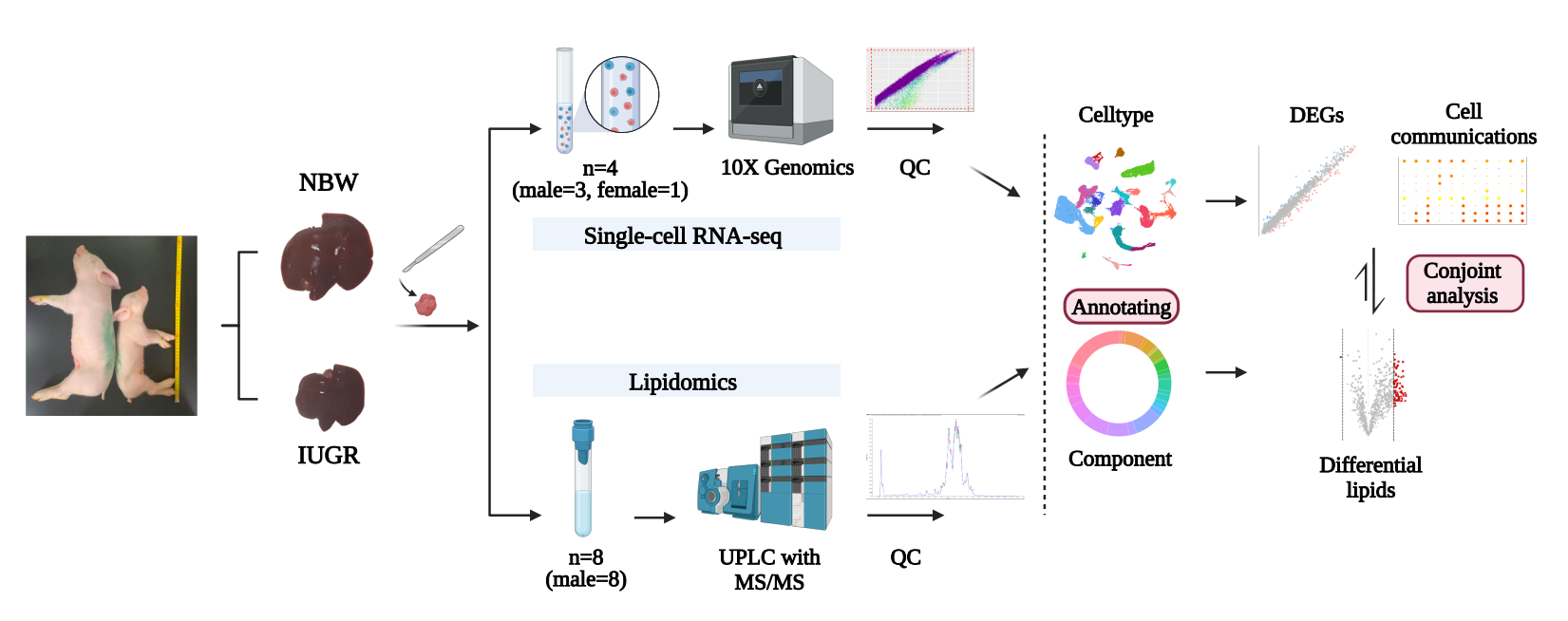
**

**Figure S4. Parameters of quality control in scRNA-seq.** a-b) Viable cells were observed in different parameters (a) and critical standards (b). c) Signature genes of each cluster on UMAP plots.

**
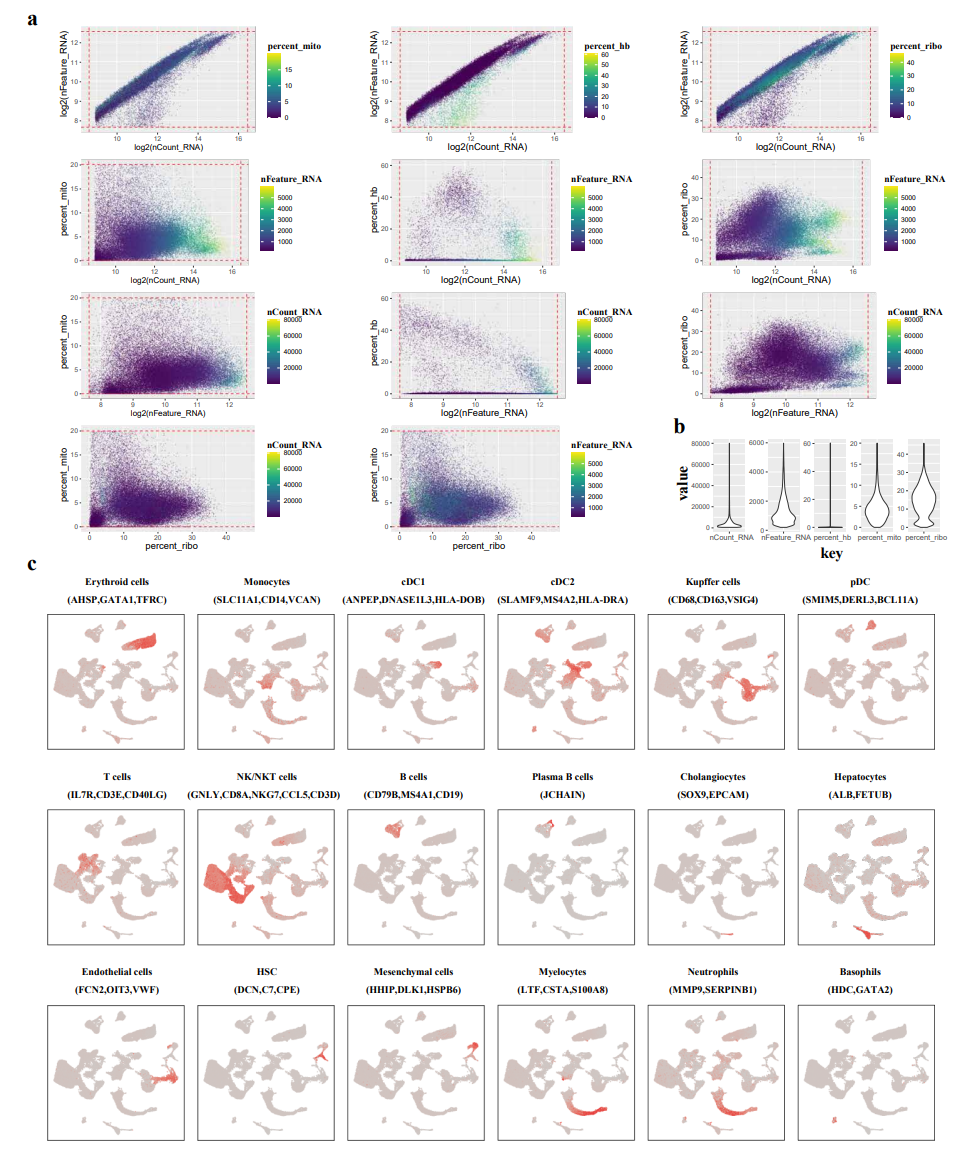
**

**Figure S5. Sexually dimorphic alterations in hepatic landscape caused by IUGR.** a-b) Cells profiled on the UMAP plots of female (a) and male piglets (b) split by IUGR and NBW. c-d) Alterations in cell ratio of the liver in female (c) and male (d) piglets experiencing IUGR.

**
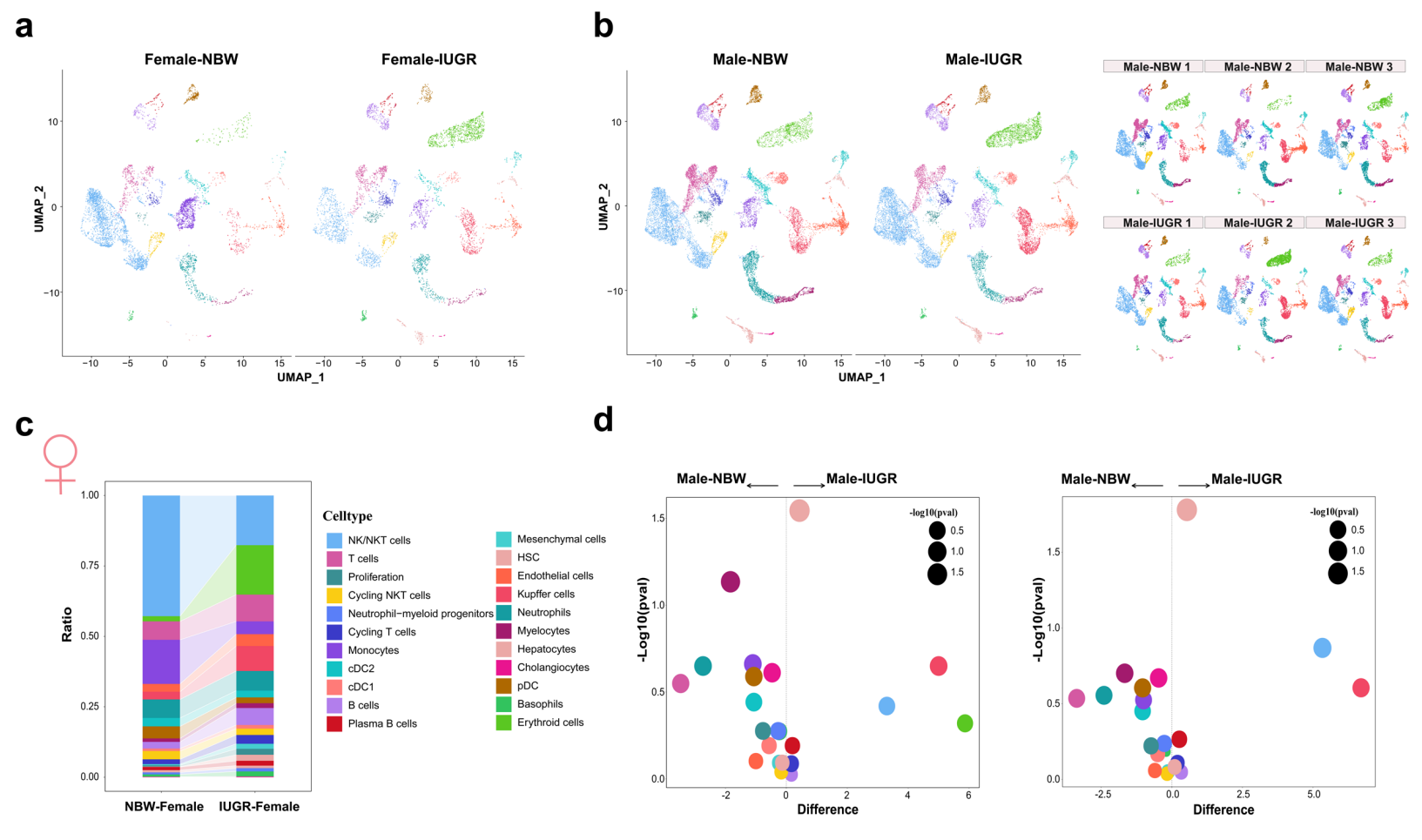
**

**Figure S6. Sexually dimorphic alterations in hepatic homeostasis caused by IUGR.** a-b) Transcriptional profiles (a) and GO functional enrichment analysis (b) of major cell types changing between IUGR and NBW in female piglets. c-d) Transcriptional profiles (c) and GO functional enrichment analysis (d) of major cell types changing between IUGR and NBW in male piglets.

**
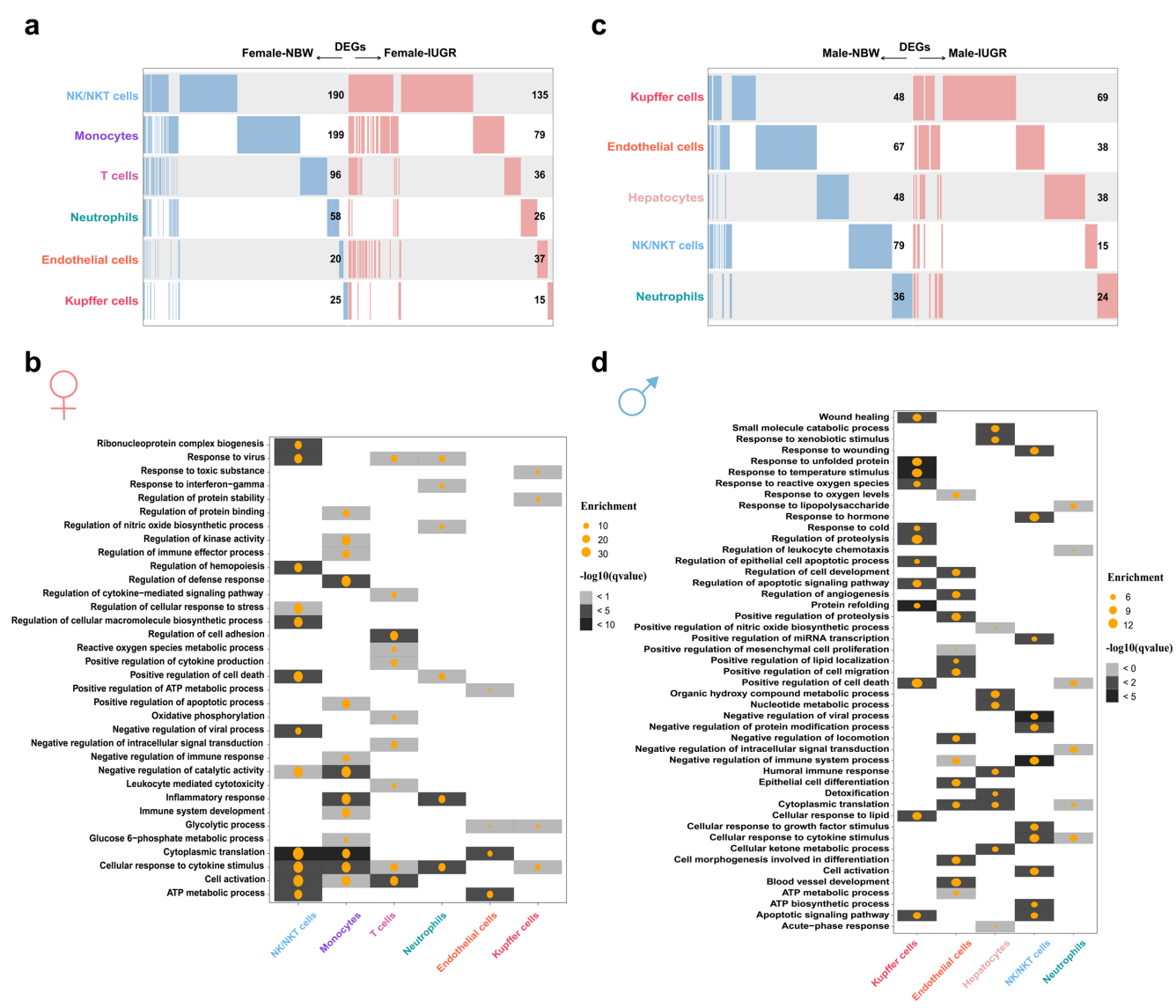
**

**Figure S7. Subclusters and pseudotime trajectory of T cells in females.** a) UMAP plots of the main subclusters of lymphoid cells in females. b) Signature gene expressions in dot plots across the CD4+ T cell subclusters. c) Panel showing the expression trajectory along pseudotime for critical genes. d) Heatmap displaying genes by BEAM analysis with notable dynamic changes and annotated GO term.

**
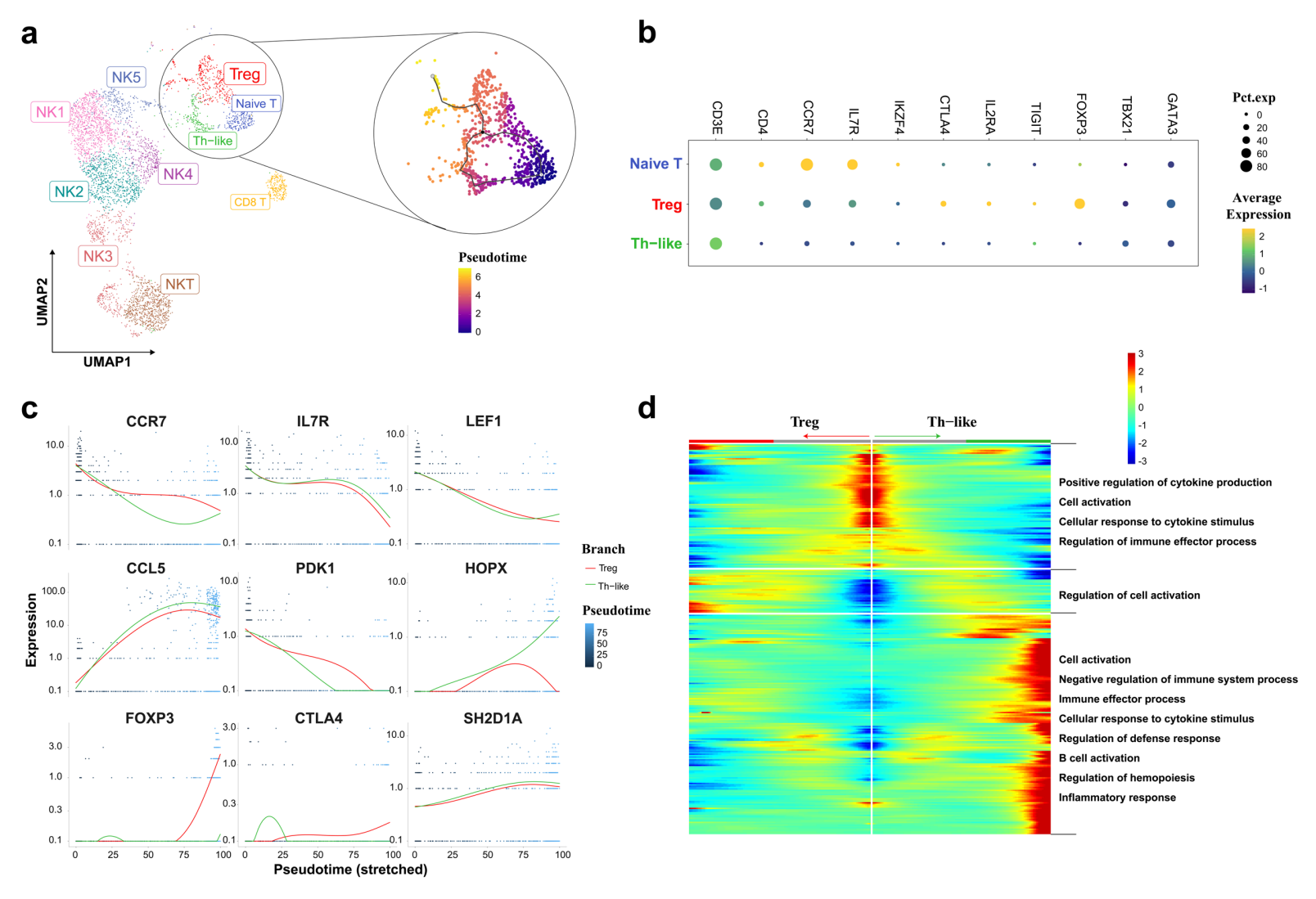
**

**Figure S8. Ecological niches of myeloid cells in the hepatic communication network in males.** a-b) Capacity for intercellular communication in the livers of NBW (a) and IUGR (b) males. c) Overview of selected ligand-receptor interactions of Kupffer cells between NBW and IUGR males. d) Pseudotime trajectory of myeloid cells in males. e) Fitting curve diagram showing the expression scores for TNFα signaling via NFκB and glycerolipid metabolism between NBW and IUGR females.

**
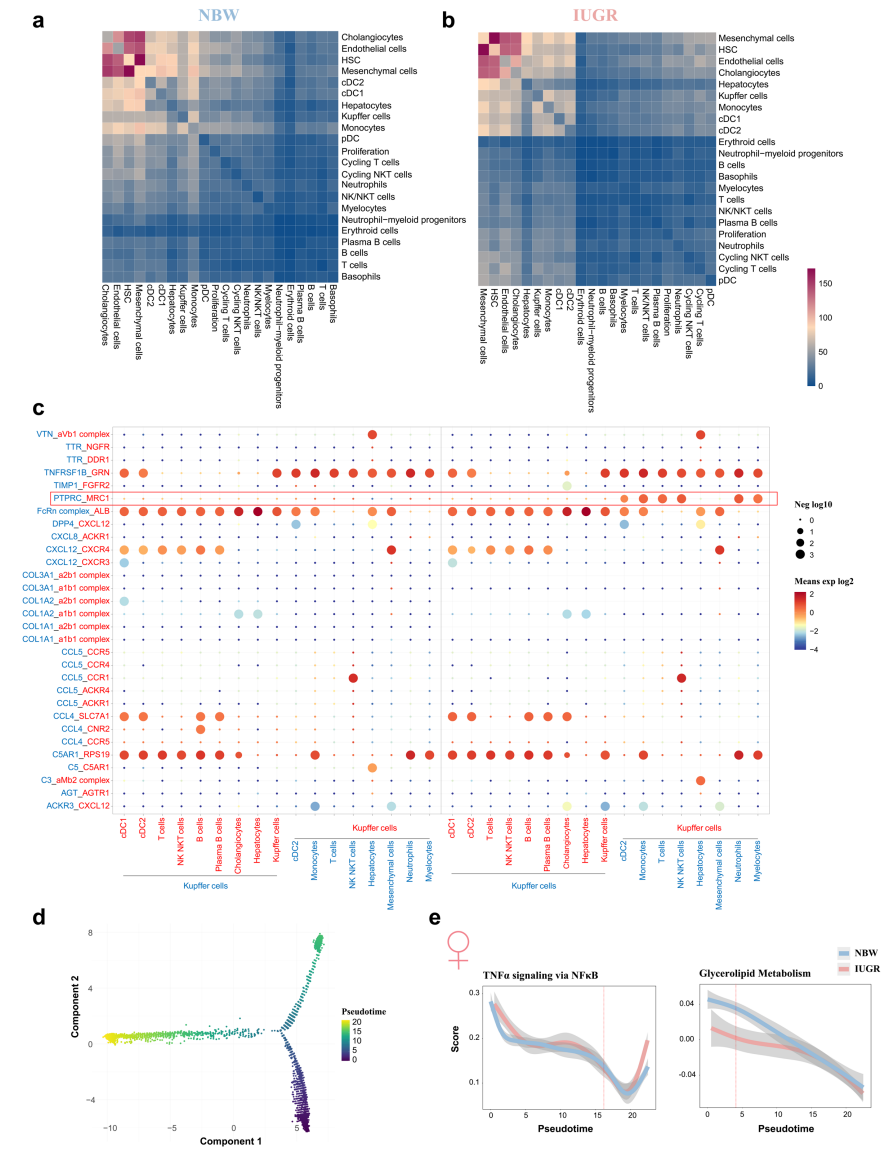
**

**Figure S9. Subclusters of Kupffer cells revealed excessive inflammation in IUGR males.** a) UMAP plots showing three subtypes of Kupffer cells identified by NNMF analysis and functional enrichment of three factors (right panel). b-c) Plots showing the normalized expression levels of specific genes (b) and pro-inflammatory score, anti-inflammatory score between inflammatory and non-inflammatory subsets and pie charts showing the contribution of IUGR and NBW males to each subset (c). d) Trajectory along pseudotime and RNA velocity vector projection of subtypes of Kupffer cells on UMAP plots. e) Heatmap revealing the dynamic changes of subtypes of Kupffer cells in gene expression based on RNA velocity. f-g) Differences in pathway activities scored by GSVA (f) and scatter plot showing DEGs (g) per cell in the inflammatory subtype between IUGR and NBW males. h) Transcriptional factor analysis on inflammatory and non-inflammatory subtypes in males. i) Rank for regulons in inflammatory subtype between IUGR and NBW males based on regulon specificity score. j) Binarized regulon activity scores of PRDM1 between IUGR and NBW males on UMAP plot. k) SEEK co-expression result for studies about “hepa” and “macro”, with regulon PRDM1 in different GEO datasets. Unpaired Wilcoxon rank-sum test (c) (*****P* < 0.0001).


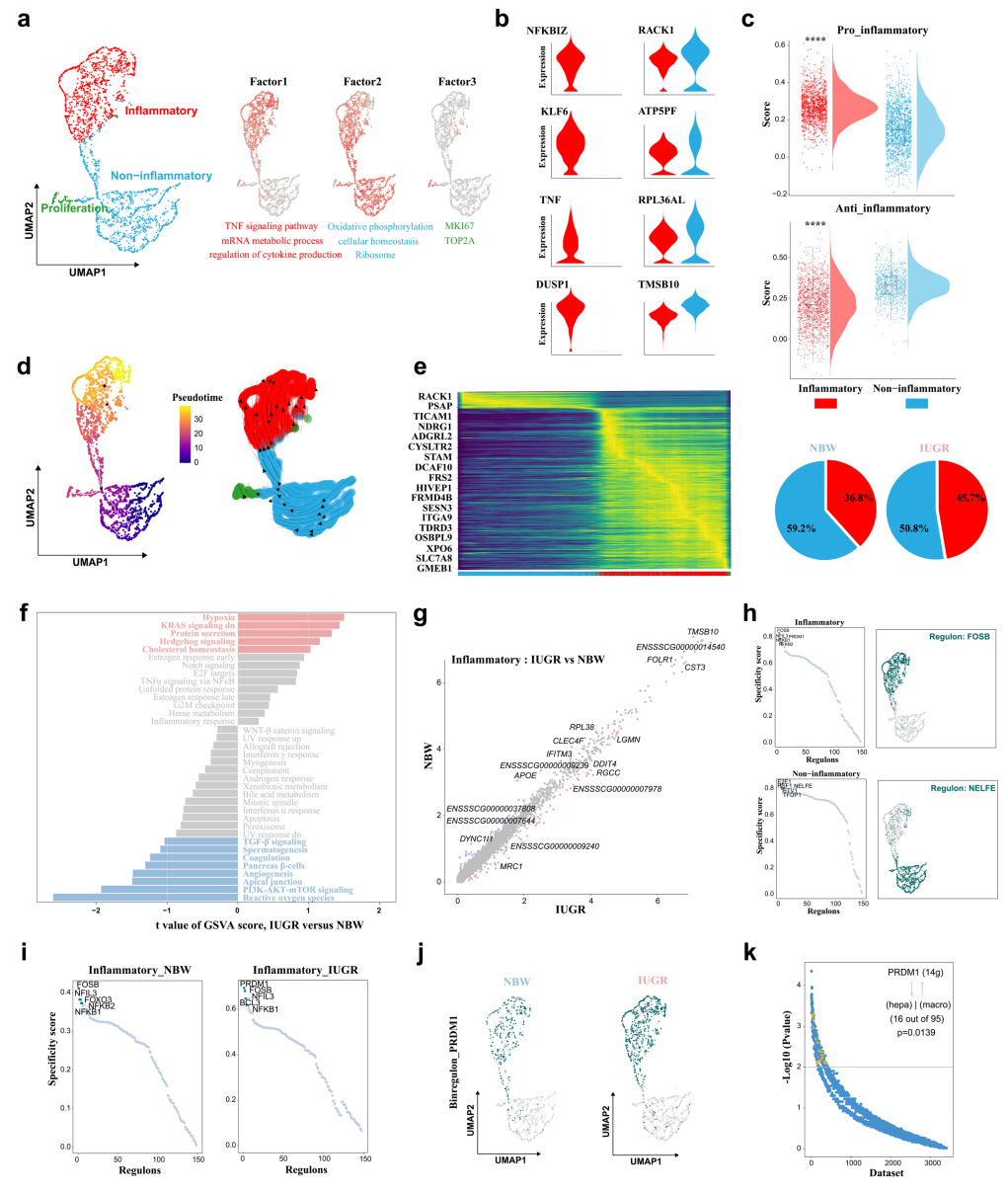


**Figure S10. Heterogeneity of non-immune cells in male livers.** a) UMAP plots showing the clustering of non-immune cells. b) Heatmap showing the expression of specific genes across non-immune cell subclusters. c) The proportion of non-immune cell subsets between NBW and IUGR males. d) Radar plots (left), example genes (middle) and selected GO enrichment (right) of metagene signatures 1-6, showing the distribution of signature expression across Endo subpopulations. Endo, endothelial; HSC, hepatic stellate cell; VSMC, vascular smooth muscle cell.


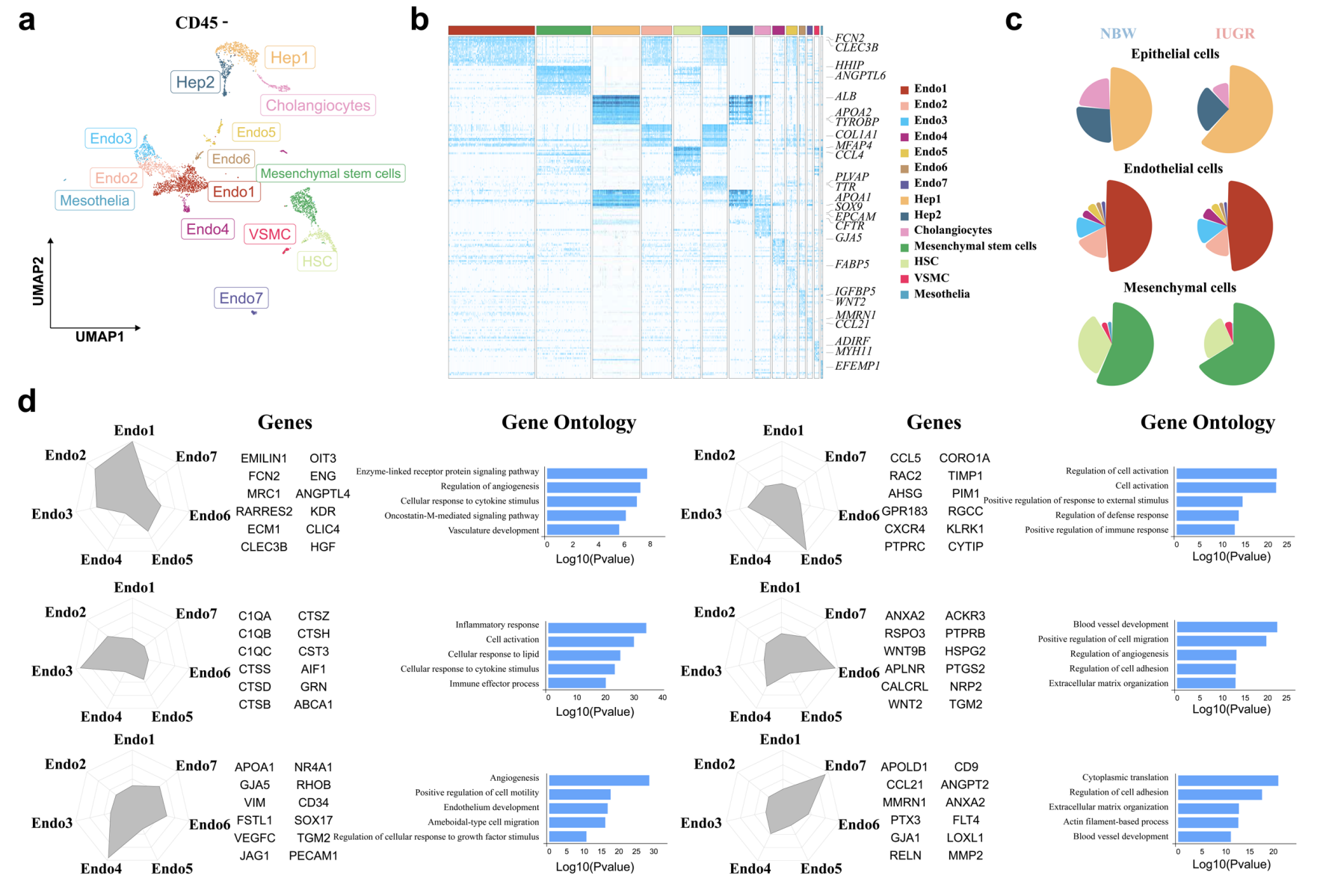


**Figure S11. Specific differential signaling pathways between IUGR and NBW males.** a-b) The role of network analysis in SCT (a) and GDF (b) pathways in NBW males. c-d) Comparison of TGF-β network (c) and L-R pair (d) between IUGR and NBW males. e-f) Difference of L-R pair (e) and expression of each cluster (f) in IGF signaling pathway between IUGR and NBW males.

**
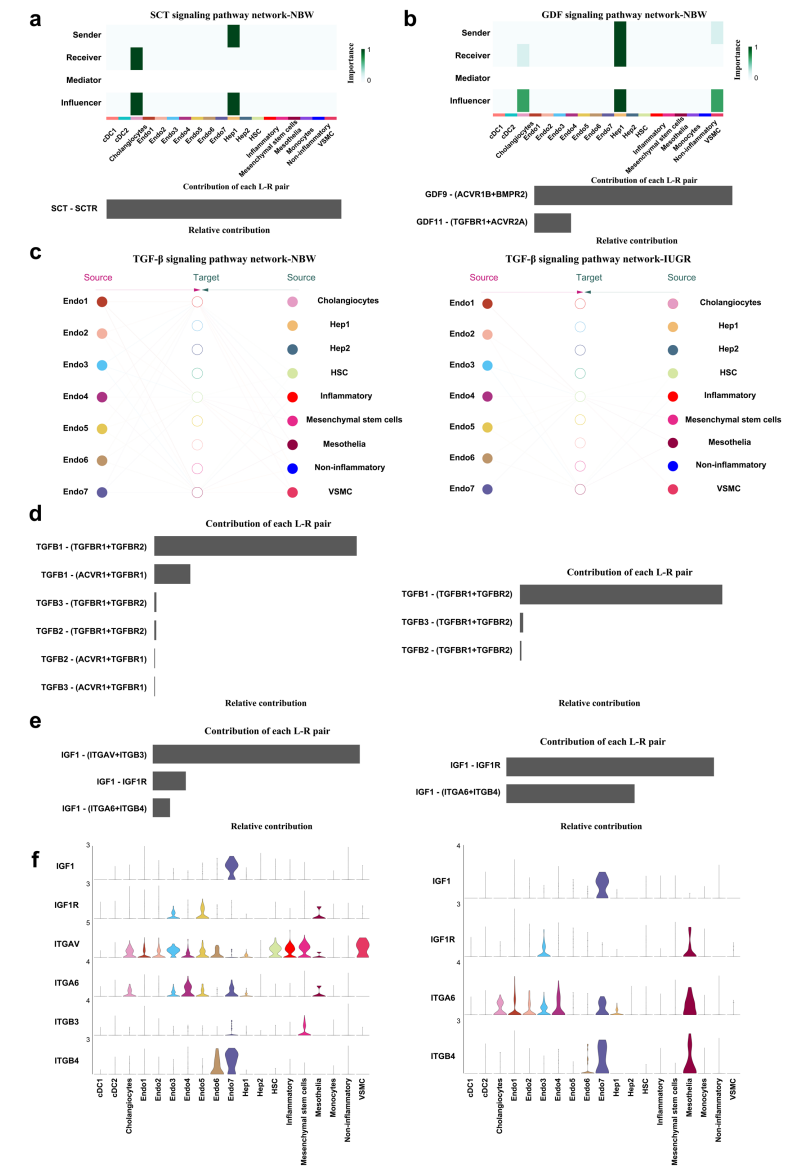
**

**Figure S12. Hepatocellular injury caused by IUGR in males.** a) The characteristics of quality control between subtypes of Hep, and GO enrichment of upregulated-genes or downregulated-genes in Hep1 between IUGR and NBW males. b) Histopathology of the liver assessed by H&E and PAS between NBW and IUGR males. c) Histopathology of the liver assessed by H&E, PAS, Oil Red O staining and TEM and between NBW and IUGR females. Scale bar, 200μm, 100μm and 2μm.


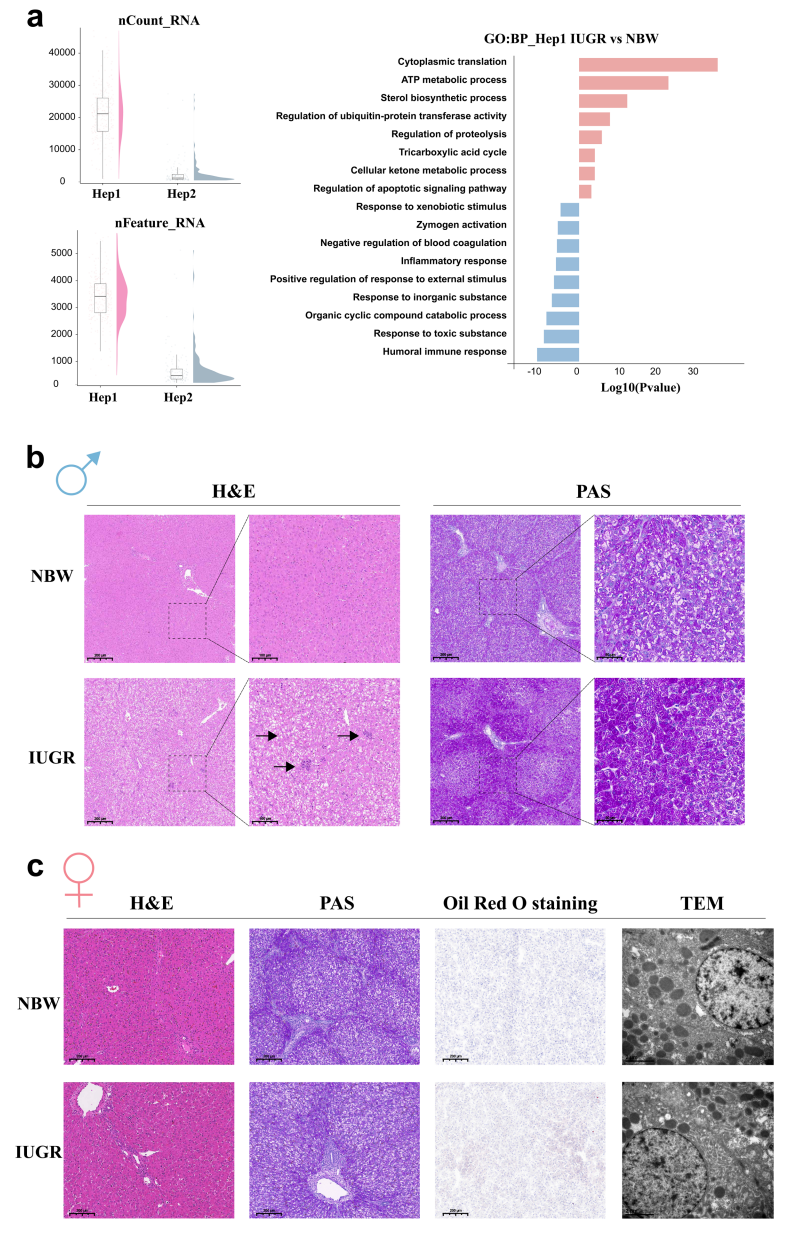


**Figure S13. Overview of lipidomics between IUGR and NBW males.** a-b) Coefficient of variation (a) and correlation (b) in QC samples. c) Pie charts of lipids identified in the MRM mode. d) Differential lipidomic patterns between IUGR and NBW males by OPLS-DA score plot. e-f) Scatter chart showing lipids sorted by content (e) and log2FC (f). g) Z-score plot showing profile of differential lipids between IUGR and NBW males. h) Functional enrichment of differential lipids. i) Correlation of differential lipids. j) Chain length and saturation of differential TGs. TG, triglyceride; PC, phosphatidylcholine;; PE, phosphatidylethanolamine; CAR, carnitine; PI, phosphatidylinositol; LPC, lyso-phosphatidylcholine; SM, sphingomyelin; FFA, free fatty acid ; Cer, ceramides; DG, diglyceride; PG, phosphatidylglycerol; PS, phosphatidylserine; LPE, lyso-phosphatidylethanolamine; PA, phosphatidic acid; HexCer, hexosylceramides; BMP, Bis(monoacylglycero)phosphate; LPG, lyso-phosphatidylglycerol; BA, bile acid; CE, cholesterylesters; LPS, lyso-phosphatidylserine; LPI, lyso-phosphatidylinositol; PMeOH, phosphatidylmethanol; SPH, sphingosine; LPA, lyso-phosphatidic acid; CerP, phosphatidylglycerol; Cert, phytosphingosine; MG, monoglyceride; CoQ, coenzyme; MGDG, monogalactosyldiacylglycerol.





**Figure S14. Overview of the expression levels of genes about the apolipoprotein family in IUGR males.** a) Violin plots showing the expression levels of genes about the apolipoprotein family in Hep1 between IUGR and NBW males. b) The foldchange of genes about the apolipoprotein family between IUGR and NBW males using RNA-seq. c) The FMPK value of APOA4 between IUGR and NBW males and females using RNA-seq. d) Comparison of the mRNA expression of APOA4 in the liver between IUGR and NBW males using RT-qPCR (n=8). d) Dot plot showing the expression of APOA4 among cell types in male liver. e) Comparison of APOA4 gene homology among multiple species. Unpaired Wilcoxon rank-sum test (a) and two-sided paired Student’s t-test (c) (*****P* < 0.0001; ***P* < 0.01; **P* < 0.05; ns *P* > 0.05).

**
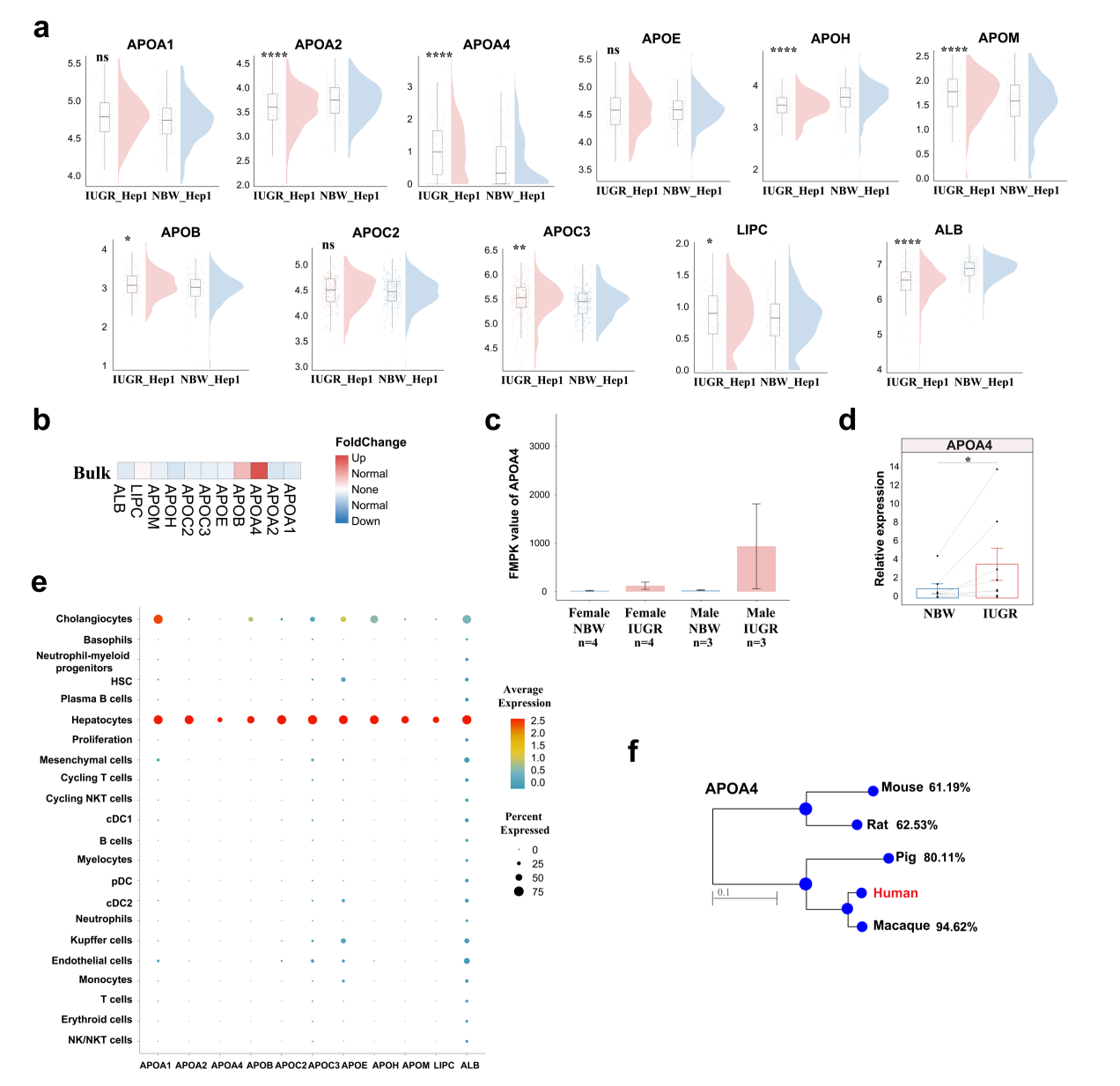
**

**Figure S15. Functional alterations about the absence of APOA4 in the HepG2 cell line.** a) Demonstration of the APOA4 editing region and gel electrophoresis images of products based on specific primer pairs. b) Verification of an APOA4-KO HepG2 cell line by sequencing. c) Images of clonogenicity and cell cycle comparison between HepG2 and APOA4-KO. d) Volcano plot showing differential gene expression between HepG2 and APOA4-KO during hypoxia condition, and functional enrichment analyses using GSVA.





**Figure S16. High-precision views of the pathology section in the liver.** a Histopathology of liver assessed by H&E and Perls stain across groups. Scale bar, 100μm and 200μm.


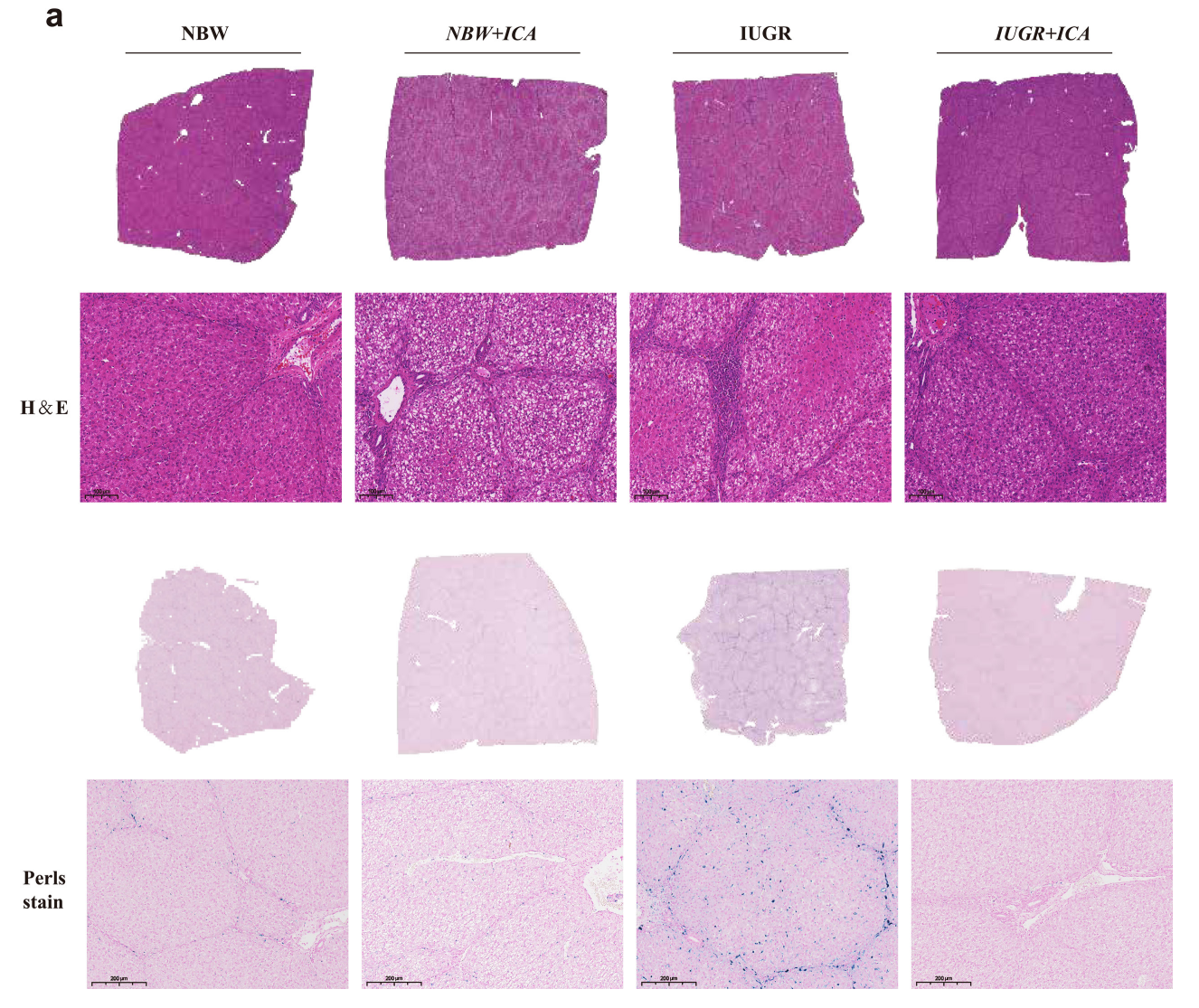


**Figure S17. Proposed scheme summarizing the study** created with BioRender.com**.** IUGR livers experiencing hypoxia were more prone to severe injury in males than in females one week after birth. In IUGR females, the fibroblasts might be activated by E2 to drive the differentiation of Treg cells, alleviating inflammation. This regulatory process improved non-immune cellular communication networks, promoting liver regeneration. In contrast, in IUGR males, the dysfunction of mitochondria impaired the oxidation of FFA and hepatocytes reduced their capacity of transportation, leading to increased synthesis and TG deposition. The accumulation of TG recruited more Kupffer cells, which triggered inflammation via PRDM1. Elevated pro-inflammatory cytokines disrupted non-immune cellular communication networks, weakening liver regeneration that persisted into adulthood. In response to these challenges in IUGR males, a protective mechanism involving upregulation of APOA4 to alleviate the burden of TG deposition through the PPAR signaling pathway was identified. E2, estradiol. Hep, hepatocytes; Endo, endothelial cells; KCs, Kupffer cells. Mes, mesenchymal stem cells. Cho, cholangiocytes.


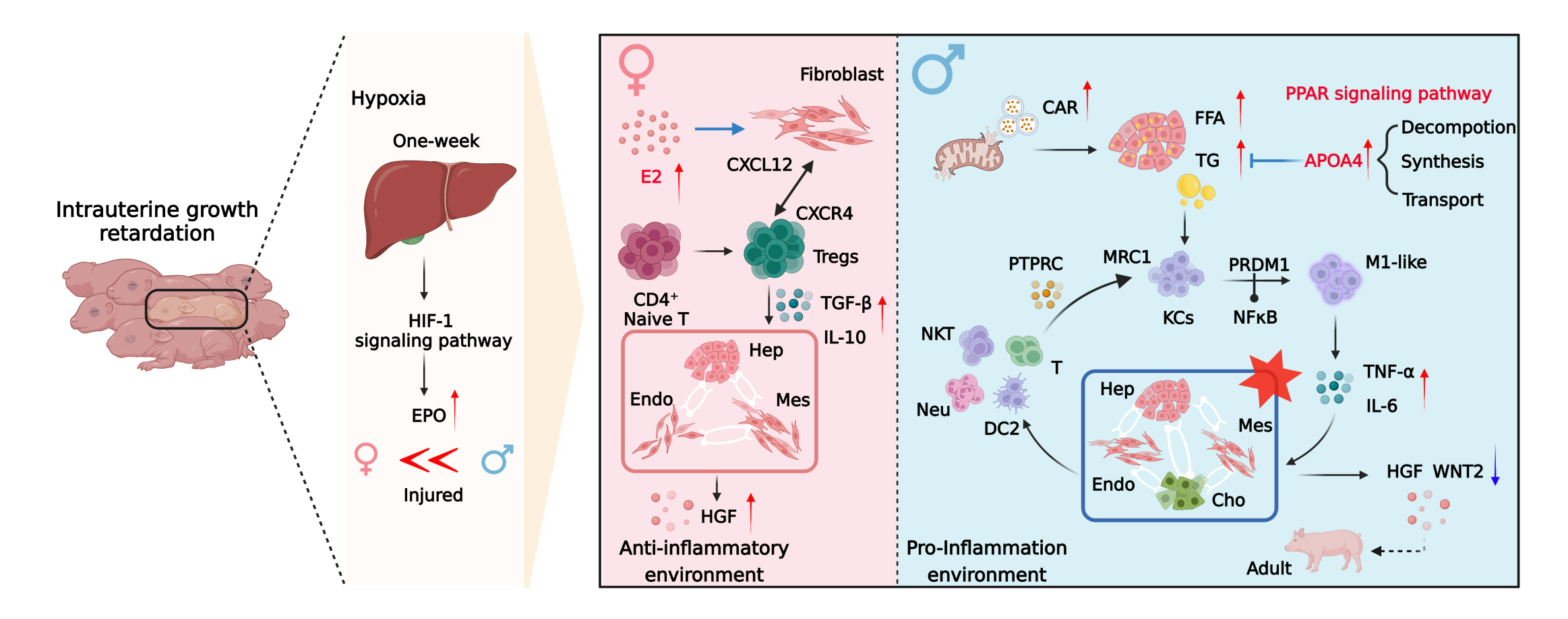


**Table S1.** Neonatal information and serum biochemical parameters related to liver function.

**Table S2.** Gene name replacement and description of previous studies on the RNA-seq on GSE106512 and PRJNA597972, and lists of differentially expressed genes.

**Table S3.** Description of scRNA-seq and differentially expressed genes in each cell type.

**Table S4.** Detailed information and clustering of regulons in all clusters generated by pySCENIC.

**Table S5.** List of DEGs on each cell type and GO enrichment of DEGs related to females suffering from IUGR in major cell types.

**Table S6.** List of DEGs on each cell type and GO enrichment of DEGs related to males suffering from IUGR in major cell types.

**Table S7.** Clustering and GO enrichment of genes related to pseudotime BEAM analysis in female T cell subtypes.

**Table S8.** Clustering and KEGG enrichment of genes related to pseudotime analysis in male myeloid cells.

**Table S9.** Top 50 genes in each factor by NMF analysis and differentially expressed genes from male inflammatory subtype.

**Table S10.** Detailed information and activity score of regulons from male inflammatory subtype generated by pySCENIC.

**Table S11.** Differential gene expression of each cell type in male non-immune cells.

**Table S12.** GO enrichment of gene signatures from endothelial subtypes.

**Table S13.** Detailed information on cell-cell communication network among male all cell types or between myeloid cells and non-immune cells.

**Table S14.** Detailed profiles of lipidomics and differential lipids between IUGR and NBW males.

**Table S15.** Description and expression matrix of RNA-seq on HepG2 and APOA4-KO under hypoxia.

**Table S16.** List of gene signatures including M1 and M2 polarization, Pro_and anti_inflammatory, Glycerolipids metabolism, TNF-NFκB signaling, and Endo signature 1-6.

**Table S17.** List of materials and methods.
